# Non-adaptive Evolution of Trimeric Autotransporters in *Brucellaceae*

**DOI:** 10.3389/fmicb.2020.560667

**Published:** 2020-11-12

**Authors:** Mohammad Reza Rahbar, Mahboubeh Zarei, Abolfazl Jahangiri, Saeed Khalili, Navid Nezafat, Manica Negahdaripour, Yaser Fattahian, Amir Savardashtaki, Younes Ghasemi

**Affiliations:** ^1^Pharmaceutical Sciences Research Center, School of Pharmacy, Shiraz University of Medical Sciences, Shiraz, Iran; ^2^Applied Microbiology Research Center, Systems Biology and Poisonings Institute, Baqiyatallah University of Medical Sciences, Tehran, Iran; ^3^Department of Biology Sciences, Shahid Rajaee Teacher Training University, Tehran, Iran; ^4^Department of Pharmaceutical Biotechnology, School of Pharmacy, Shiraz University of Medical Sciences, Shiraz, Iran; ^5^Department of Biotechnology, Institute of Science and High Technology and Environmental Sciences, Graduate University of Advanced Technology, Kerman, Iran; ^6^Department of Medical Biotechnology, School of Advanced Medical Sciences and Technologies, Shiraz University of Medical Sciences, Shiraz, Iran

**Keywords:** *in silico*, bioinformatics, TAA, BatA, *Brucellaceae*, adhesin

## Abstract

*Brucella* species are Gram-negative, facultative intracellular pathogens. They are the main cause of brucellosis, which has led to a global health burden. Adherence of the pathogen to the host cells is the first step in the infection process. The bacteria can adhere to various biotic and abiotic surfaces using their outer membrane proteins. Trimeric autotransporter adhesins (TAAs) are modular homotrimers of various length and domain complexity. They are a diverse, and widespread gene family constituting the type Vc secretion pathway. These adhesins have been established as virulence factors in *Brucellaceae*. To date, no comprehensive and exhaustive study has been performed on the trimeric autotransporter family in the genus. In the present study, various bioinformatics tools were used to provide a novel evolutionary insight into the sequence and structure of this protein family in *Brucellaceae*. To this end, a dataset of all trimeric autotransporters from the *Brucella* genomes was built. Analyses included but were not limited to sequence alignment, phylogenetic tree constructions, codon-based test for selection, clustering of the sequences, and structure (primary to quaternary) predictions. Batch analyzes of the dataset suggested the existence of a few structural domains within the whole population. BatA from the *B. abortus* 2308 genome was selected as a reference to describe the features of these structural domains. Furthermore, we examined the structural basis for the observed rigidity and resiliency of the protein structure through a molecular dynamics evaluation, which led us to deduce that the random drift results in the non-adaptive evolution of the trimeric autotransporter genes in the *Brucella* genus. Notably, the modifications have occurred across the genus without interference of gene transmission.

## Introduction

Members of the genus *Brucella* are Gram-negative, facultative intracellular pathogens of the alpha-subclass of Proteobacteria phylum. *Brucella* species cause brucellosis, a damaging zoonotic disease, in a broad range of mammalian hosts. Brucellosis imposes a significant health burden, particularly in developing countries and in the livestock dependent economies (Gopalakrishnan et al., 2016). The ingestion of contaminated dairy products and direct contact with the infected animals could transmit the infection to humans, whereas infection in animals occurs through the environmental transmission of bacteria (Gopalakrishnan et al., 2016).

The typical species within the *Brucellae* genus share a high level of genomic identity. This observation initially led to the conclusion that *Brucella* is a monospecific genus (*B. melitensis*), which also contained six biovars based on their host prevalence. However, recent molecular analyses suggest that *B. abortus, B. melitensis, B. neotomae*, and *B. ovis* are four related clones of one organism; whereas, *B. suis* forms a separate cluster (Foster et al., 2009; Whatmore, 2009). Currently, at least 12 *Brucella* species are identified (El-Sayed and Awad, 2018). The most economically significant species are *B. abortus*, *B. melitensis*, and *B. suis*.

The *Brucella* ancestor was a free-living bacterium that became an animal parasite (El-Sayed and Awad, 2018). Some species have only one chromosome. Most species, by contrast, harbor two chromosomes; one larger in size and a smaller one originating from a plasmid. Interestingly, some species such as *B. suis* have also kept their ancestor accessory genes, responsible for exploiting plant-derived nutrients. *B. suis* chromosome 1 and the genome of *Mesorhizobium loti*, a plant symbiont, show a high level of gene synteny. Their metabolic activities are similar to those of soil-plant associated bacteria, suggesting an evolutionary relationship between *Brucellae* and the plant pathogens and symbionts (Paulsen et al., 2002; Ficht, 2010).

The bacterial attachment to some molecules on the host cells triggers a multistep infection process, which is followed by internalization, replication, and survival within macrophages. A delay in an antibiotic-based treatment following the initial stages of the infection could ultimately lead to the settlement of *Brucellae* in various organs and tissues (Corbel, 1997). The progress in the course of infection demands several mechanistic determinants and regulatory systems. Professional and non-professional phagocytic cells provide a replication niche for the bacterium; they can also carry the pathogen through the mucosal surfaces into the lymph nodes (Roop et al., 2009).

The secretome and membrane proteome of the pathogens are an integral part of interaction between bacterial cells and their environment. Bacteria can sense stress, adhere to various biotic and abiotic surfaces, and establish a cell to cell communication by means of some secreted proteins (Sankarasubramanian et al., 2016a).

Adherence to the host cells governs the cellular and tissue tropism, entrance site, host specificity, and replicative niche of the pathogen (Czibener and Ugalde, 2012). The pathogen’s outer membrane proteins play pivotal roles in this process (Edmonds et al., 2001). Several open reading frames, coding for different adhesin proteins, have been found in *Brucella* genomes (Rocha-Gracia et al., 2002; Castañeda-Roldán et al., 2006; Backert et al., 2008; Posadas et al., 2012; Czibener et al., 2016; Sankarasubramanian et al., 2016a). A pathogenicity island harboring an immunoglobulin-like domain protein was also identified in the *Brucellae* genomes. This domain involves the attachment and internalization of the bacteria into the host cells (Czibener and Ugalde, 2012). More recently, in *Brucella*, a collagen-binding adhesin (major outer membrane protein, Bp26) was described by enzyme-linked immunosorbent assay (ELISA) and bio-layer interferometry. The protein was shown to bind to type I collagen, vitronectin, and soluble fibronectin (ElTahir et al., 2019).

Two members of trimeric autotransporter adhesins (TAAs, also known as type Vc secretion pathway) namely, BtaE (Ruiz-Ranwez et al., 2013b), and BtaF (Ruiz-Ranwez et al., 2013a) were identified in *B. suis*. Both adhesins involve the attachment of bacteria to the extracellular matrix. Functional studies using genetic knockouts of the adhesin proteins reduce the virulence of *B. suis*.

The comparative genome analysis of *B. abortus* 2308 and *Brucella* S19, a spontaneously attenuated vaccine strain, resulted in the identification of a set of virulence genes. One of the candidates for virulence is BAB1_0069 (Crasta et al., 2008), which, encodes an ortholog of BtaE. An adherence assay suggested that this adhesin participates in the interaction of *B. abortus* with the host cell, similar to its ortholog in *B. suis* 1330 (Ruiz-Ranwez et al., 2013b). This ortholog also assists the initial attachment of the bacteria to the HeLa cells (Sieira et al., 2017). The corresponding locus in S19 has undergone a 1,695-nucleotide deletion. Hence, 565 amino acids were removed from the mature protein during the deletion, so that the attenuated S19 strain comprises a 768 aa long adhesin. Similar deletions were also observed in the same open reading frame of other virulent species. In *B. melitensis*, the locus contains three deletions: the first causes a 512 amino acid deletion, second and third are single nucleotide deletions leading to a frameshift. As a result, the strain contains only the 365 C-terminal amino acids. In *B. suis* genome, the related open reading frames are predicted to encode a 740 amino acid long protein because of two deletions. The position and length of these open reading frames are identical in the attenuated S19 strain and the virulent *B. suis.* The difference is related to 133 amino acid repeat module. These deletions and similar ones in other virulent species have ruled out the association of deletions with the lack of virulence in the S19 strain (Crasta et al., 2008). Therefore, the trimeric autotransporters are putative virulence factors in *Brucellae* and not essential or key virulence factors.

Trimeric autotransporter adhesins are a diverse, simple, and widespread type Vc secretion pathway (Dautin and Bernstein, 2007; Bernstein, 2019; Kiessling et al., 2020). TAAs are modular homotrimers with various lengths and domain complexity. The structure of these adhesins obeys the simple pattern of (from the N-terminal) signal peptide, passenger, and membrane anchoring domain (Linke et al., 2006). The passenger is a complex of structurally-conserved analogous blocks, which immediately forms a trimeric quaternary structure upon passing through the barrel made by membrane anchoring domain (Linke et al., 2006). The rearrangement of these blocks has resulted in considerable diversity among family members.

The TAAs are well-worthy of study as potential candidates to design novel vaccines, disease biomarkers, and novel anti-virulence drugs (Qin et al., 2015). Understanding the intricacies of their structure, architecture, and evolutionary insights are critical for developing novel therapeutic approaches and to advance the field further.

In recent years, several members of TAAs have been investigated in extensive detail. As the crystal structures of various domains of TAAs are solved, it becomes feasible to identify the domains of new members via a homology modeling-based approach. The examples of solved crystal structures are the crystal structure of *Burkholderia pseudomallei* antigen Bpsl2063 under Protein Data Bank (PDB) accession number 4USX (Gourlay et al., 2015), *Acinetobacter* species (sp.) Tol 5 AtaA C-terminal stalk (PDB ID: 3WPA; Koiwai et al., 2016), *Salmonella enterica* SadA coiled-coil domain (PDB ID: 2WPQ; Hartmann et al., 2009), crystal structure of Hia binding domain from *Haemophilus influenzae* genome (PDB ID:1S7M; Yeo et al., 2004); structure of the head of the *Bartonella* adhesin BadA (PDB ID: 3D9X; Szczesny et al., 2008); and the left-handed parallel beta-roll collagen-binding domain of *Yersinia enterocolitica* adhesin YadA (PDB ID: 1P9H; Nummelin et al., 2004).

In the present study, a wide variety of *in silico* tools were employed to determine the properties of trimeric autotransporters in *Brucella* species (BTAA). This paper provides comprehensive information, regarding the distribution of TAAs in the genus, the architecture of BTAAs, and a hypothesis as to the evolution of the BTAAs. BTAA sequences were extracted from databases to make a dataset. Batch analyzes of the dataset suggested the existence of a few structural domains. Focusing on TAA from the *B. abortus* 2308 genome enabled us to describe some features of BTAAs. Henceforth, these proteins were described as BatA (*Brucella abortus* trimeric autotransporter adhesin). Following homology modeling, the sequence and structural features of the proteins were annotated. The evolutionary analysis were also performed and showed that these domains are repeated by different frequencies within BTAA sequences. We finally concluded that the random drift results in the non-adaptive evolution of TAA genes in the *Brucella* genus.

## Materials and Methods

### Data Sources

The phylogenetic tree of *Brucellaceae*, the related genomes, protein sequences, and protein-coding genes were obtained from the Pathosystems Resource Integration Center (PATRIC ver. 3.5.39; Wattam et al., 2016). The BLAST databases were built separately from genomes, proteomes, and protein-coding genes of all members of the *Brucellaceae* family using the makeblastdb application version 2.9.0 (Altschul et al., 1997) in the UGENE environment version 1.32 (Okonechnikov et al., 2012). For all BLAST search strategies, the consensus sequence of YadA like C-terminal domain (IPR005594) was queried. The significant BLAST hits were mapped to related genomes on the phylogenetic tree of the *Brucellaceae* family.

### Evolutionary Analysis

The evolutionary analyzes including disparity index test, detecting duplication events, alignments, and phylogenetic tree constructions were performed in MEGA 7 software (Kumar et al., 2016). All analyses were based on the nucleotide sequences of the highly conserved membrane anchoring region of BTAAs; and the sequences were grouped based on related species. The best evolutionary model for analyzing the DNA dataset was estimated by the model selection function of MEGA 7.

Possible recombination events were tracked by the Genetic Algorithm for Recombination Detection (GARD; Kosakovsky Pond et al., 2006) as provided by the Datamonkey server (Weaver et al., 2018) at www.datamonkey.org. The analysis was performed on the complete nucleotide sequences of BTAA encoding genes of *Brucellaceae*.

Codon bias within the nucleotide sequences of BTAAs was inferred by MEGA 7. Codon usage of each representative sequence was compared to the species codon table by the graphical codon usage analyzer (Fuhrmann et al., 2004) as provided by http://www.gcua.schoedl.de. The codon usage frequency was converted into relative adaptiveness values. Contrary to the codon usage frequency, the relative adaptiveness takes into account the number of codons which code for the respective amino acid. For each amino acid, the codon with the highest frequency value was set to 100% relative adaptiveness. All other codons for the same amino acid were scaled accordingly. The codon usage table of each representative genome was obtained from the codon usage database at https://www.kazusa.or.jp (Bzhalava et al., 2018). The nucleotide sequence of Omp31, a species-specific gene (Herman and De Ridder, 1992; Romero et al., 1995; Singh et al., 2013), was used as control.

### Codon-Based Test for Selection

The probability of rejecting the null hypothesis of strict-neutrality (d_*N*_ = d_*S*_) in favor of the alternative hypotheses [positive (d_*N*_ > d_*S*_) and purifying (d_*N*_ < d_*S*_) selections] was calculated on membrane anchoring domain encoding portion of the genes in MEGA 7 based on the *Z*-test and Fisher’s exact test of neutrality (Zhang et al., 1997). Values of *P* less than 0.05 were considered significant. The variance of the difference was computed using 1000 bootstrap replicates. The analyses were conducted using the Pamilo-Bianchi-Li method (Pamilo and Bianchi, 1993) (d_*N*_: non-synonymous substitutions per non-synonymous site; d_*S*_: synonymous substitutions per synonymous site).

### Clustering the BTAA Sequences

An all-against-all approach was employed for clustering the amino acid sequences of all TAAs from *Brucella* genomes by CLANS software as provided by the MPI Bioinformatics Toolkit (Zimmermann et al., 2018) at https://toolkit.tuebingen.mpg.de (Alva et al., 2016). The pairwise attraction values based on the *P* values of the high scoring segment pairs (HSPs) were calculated. The BLAST analysis of each BTAA sequence against all other BTAA sequences based on the *P* values of high-scoring segment pairs was performed and enabled the three-dimensional visualization of pair-wise sequence similarities. The resulting file was visualized and analyzed by CLANS stand-alone software (Frickey and Lupas, 2004). To keep the sequences from collapsing into one point, the *P* values above 10^–6^ were discarded. Furthermore, the distribution of HSPs was collected and mapped onto the BatA sequence as a prototype and visualized in a graph.

### Primary Sequence Analysis

The domain architectures of proteins were viewed at the InterPro database (Finn et al., 2017) and Pfam (Finn et al., 2016).

#### Signal Peptide

The presence of signal peptides (SP) was assessed by Phobius (Käll et al., 2004) at http://phobius.sbc.su.se/ (Käll et al., 2007).

The location of signal peptides was predicted based on the SignalP ver. 5 (Petersen et al., 2011) server at http://www.cbs.dtu.dk, using deep neural network [taking advantage of the algorithm ability to differentiate the “standard” signal peptidase I-cleaved SPs translocated by the Sec translocon (Sec/SPI) and two other types of SPs in prokaryotes, namely lipoprotein SPs cleaved by signal peptidase II (Sec/SPII) and Twin-Arginine Translocation (Tat) SPs translocated by the Tat translocon (Tat/SPI)].

The presence and location of Twin-arginine signal peptide cleavage sites in BTAA sequences were predicted by TatP ver 1 server (Bendtsen et al., 2005) at http://www.cbs.dtu.dk using a combination of two artificial neural networks. Pred-Tat (Bagos et al., 2010), at http://www.compgen.org, was employed for predicting the signal peptides based on the hidden Markov model. The Signal BLAST server (Frank and Sippl, 2008) at http://sigpep.services.came.sbg.ac.at was also employed for comparing the signal peptide against the Uniprot database (Consortium, 2017).

#### Repeat Modules

The repeat modules within the primary sequences were identified by generating a dot-plot matrix at https://myhits.isb-sib.ch by Dotlet software (Junier and Pagni, 2000). The Xstream software (Newman and Cooper, 2007) available at http://jimcooperlab.mcdb.ucsb.edu and the T-REKS standalone software (Jorda and Kajava, 2009) were also employed for defining the repeat modules within the entire protein dataset.

#### Coiled-Coils

The existence of coiled-coil regions was predicted by the Wagga Wagga (Simm et al., 2015) server at https://waggawagga.motorprotein.de. The server employs six external tools to determine the position of coiled-coil regions.

#### Consensus Patterns

Consensus patterns were searched across the primary amino acid sequences by the ScanProsite tool at https://prosite.expasy.org (De Castro et al., 2006). The consensus patterns and motives were extracted from the literature (Szczesny et al., 2008; Leo et al., 2011; Bassler et al., 2015).

### Gene Synteny and Location of BTAAs

The gene order in the vicinity of BTAAs was assessed by the SyntTax server (Oberto, 2013) implementable at http://archaea.u-psud.fr. The 60 C-terminal residues related to the membrane anchoring region were queried to perform the analysis.

The existence of BTAAs in association with other genes to build an operon was assessed at http://www.microbesonline.org and http://operondb.cbcb.umd.edu by querying the locus tags of the protein-encoding genes.

### Structure Prediction

#### Topology Prediction

The transmembrane beta-barrel topology of the BTAAs was predicted by BOCTOPUS at http://boctopus.bioinfo.se/pred/, PRED TMBB at http://bioinformatics.biol.uoa.gr/PRED-TMBB/input.jsp and PRED TMBB2 at http://www.compgen.org/tools/PRED-TMBB2.

#### Secondary Structure Prediction

The 3-state secondary structures of the proteins were predicted at RaptorX server (Wang et al., 2016) at http://raptorx.uchicago.edu, based on an emerging machine learning model (Wang et al., 2015). The accuracy of RaptorX for 3-state secondary structure prediction is 84%. The secondary structures were also predicted by PSIPRED (McGuffin et al., 2000), available by the UCL department of computational biology at http://www.bioinf.cs.ucl.ac.uk.

### Tertiary Structure Prediction and Evaluation

#### Finding the Templates

Considering the importance of *B. abortus* 2308 and the aforesaid facts, we prompted to model the tertiary structures and forecast some other properties of these building blocks. To this end, we chose a sequence (1333 amino acid in length) from *B. abortus* 2308 (locus tag: BAB1_0069) as a prototype; the sequence is called BatA (*Brucella abortus* trimeric autotransporter adhesin) hereafter.

To achieve the best template for the 3D-structure prediction, four approaches were employed. (i) the amino acid sequence of BatA was subjected to PSI-BALST against the PDB with five iterations. The structures with lower *E*-Values (lower than 10^–4^), higher identities (more than 40%), and better query coverages (more than 40%) were selected. (ii) Searching for templates, which is also a part of tertiary structure predictor algorithms using different methods and even external softwares, such as Phyre2 (Kelley et al., 2015), SwissModel (Waterhouse et al., 2018), and I-TASSER (Yang et al., 2015). (iii) Additionally, a pairwise comparison of hidden Markov models was applied for the BatA sequence to find the remote homologs by HHpred software at www.toolkit.tuebingen.mpg.de (Söding et al., 2005). This method is more sensitive than the BLAST search for finding the remote homologous structures. HHpred can detect homologous associations far beyond the twilight zone (below 20% sequence identity). in this method, the estimated probability of the template to be homolog to the query is the most important principle for choosing the template. (iv) The models generated by the tertiary structure predictors (Phyre2, SwissModel, and I-Tasser) were also added to the collection of templates.

The structures with satisfactory results in terms of sequence identity and similarity to each part of BatA were used as the templates for homology modeling by Modeler (Webb and Sali, 2014). The approach initiated by the alignment of the templates, aligning templates with the query, then generating 10 models for each query.

In all stages, the generated models (modeled with the online software tools or Modeler) were evaluated in terms of quality and agreement between the secondary structures of the 3D models and the predicted ones. The quality of the predicted models was evaluated by MolProbity (Chen et al., 2010) at http://molprobity.biochem.duke.edu.

To obtain the quaternary structures of each part, the predicted qualified models were submitted to GalaxyHomomer (Baek et al., 2017), embedded in http://galaxy.seoklab.org server. The predicted models underwent *ab initio* docking, and after refinement, final trimers were generated. The additional refinements were done at the Galaxy server, if needed.

### Channel Analysis

The analysis of the membrane channel of the protein was based on Caver ver. 3 (Chovancova et al., 2012), embedded as a plug-in in the Pymol software (DeLano, 2002). ChexVis (Masood et al., 2015), at https://vgl.csa.iisc.ac.in, was also employed for analyzing the channel properties. The probe radius was set at zero angstroms to consider channels of any width as feasible.

### Flexibility Simulations of the Protein Structures

The flexibility and toughness of the modeled structures were assessed via simulation of protein dynamics by the CABS-Flex tool (Jamroz et al., 2013). The restraints were generated for all residues by selecting the “All” mode. The minimum distance along the protein chain for the two residues to be bound was set at 2, meaning that each residue cannot be restrained with contiguous residues. Restraints were automatically generated for residues within the minimal and maximal distances of 3.8 to 8 Angstrom (default setting length of restraints). The number of cycles was set at 100, and the temperature of the simulation was 1.4.

### Cavities in the C-Terminal Coiled-Coil

While cavities in the coiled-coils are assumed to play role in the bending properties of TAAs (Leo et al., 2011), the presence and volume of the cavities in the C-terminal coiled-coils were evaluated and measured by the CASTp server (Tian et al., 2018) at http://sts.bioe.uic.edu/castp. A similar assessment was also conducted by CavityPlus (Xu et al., 2018) at http://repharma.pku.edu.cn/cavityplus; and the overlapping results of both servers were presented. Furthermore, to better understand the effect of cavities, the volume data was compared to similar data derived from the structure of the 527–665 fragment of UspA1 protein from *Moraxella catarrhalis* (Conners et al., 2008; PDB ID: 2QIH) coiled-coil region of Escherichia *coli* EibD (PDB ID: 2XZR; Leo et al., 2011), as well as a conserved coiled-coil segment of TAA of *Y. enterocolitica* (Alvarez et al., 2010; PDB ID: 3H7X).

### Protein and Membrane Alignment

To obtain the predicted model representation in a natural-like environment, the model was embedded within a membrane patch. VMD v1.9.2 software was used to perform all steps of the process, while the Tcl scripting language was used to perform all of the external manipulations. The membrane builder tool of the VMD software was used to build a membrane patch (*X* length of 80 and *Y* length of 80). The protein was aligned according to its center of mass within the lipid bilayer. Setting ten residues of membrane barrel as the center of mass, the protein was located at a proper orientation. Moreover, the matrix was set to rotate about the *X*, *Y*, and *Z*-axis by 0, −90, and 0. All of the overlapping lipid molecules with the protein stem were removed for the proper accommodation of the protein within the membrane. Water molecules were also added to the protein and membrane structure using the VMD solvate tool.

## Results

### Genomic Context of BTAAs, No Reliable Evidence of Operon Arrangement

**FIGURE 1:**
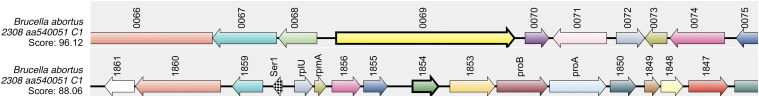
The location of the BatA encoding gene in the genome of *B. abortus* 2308. Different loci are presented with arrows of different colors. The locus tags are at the top of each arrow. BAB1_0069 and BAB1_1854 are trimeric autotransporter encoding genes.

To observe the location of BTAA encoding genes and their neighbors, a gene synteny analysis was performed. In all *Brucella* species, the TAA encoding genes are located in chromosome 1; the immediate neighbor of the TAA encoding genes is the invasion-associated locus B [in the case of *B. abortus* 2308, TAA (BatA, Locus tag: BAB1_0069), which is located 209 nucleotide pairs downstream of the invasion-associated locus B (Ialb, locus tag: BAB1_0070)] (Figure 1). This pair of genes are located in the same direction, making a set of consecutive genes on the same DNA strand.

To determine whether *batA* is associated with other genes, the operon databases were searched for related locus tags. Based on the operon database, the probability of existence of *batA* and its downstream neighbor in the same operon is estimated as 0.80 (average). This indicates that these genes are likely to be parts of the same operon. Values near to 1 or 0 are confident predictions of being in the same operon or not, respectively, while values near 0.5 are low-confidence predictions. Searching the operon database also indicated that these two genes could be found near each other in the genomes of few genera and species including *Bartonella*. The confidence was approximately 42 percent, which is a relatively low score. The conservation of this nucleotide pair across multiple genomes was estimated as 0.75, based on Microbesonline Ortholog Groups Dataset. The conservation score here is typically ranged from 0 (not conserved) to 1 (100% conserved). Based on these results, BTAAs encoding genes and their neighbors do not arrange as an operon.

### Two Trimeric Autotransporters Are Present in *B. abortus* 2308 Genome

**FIGURE 2:**
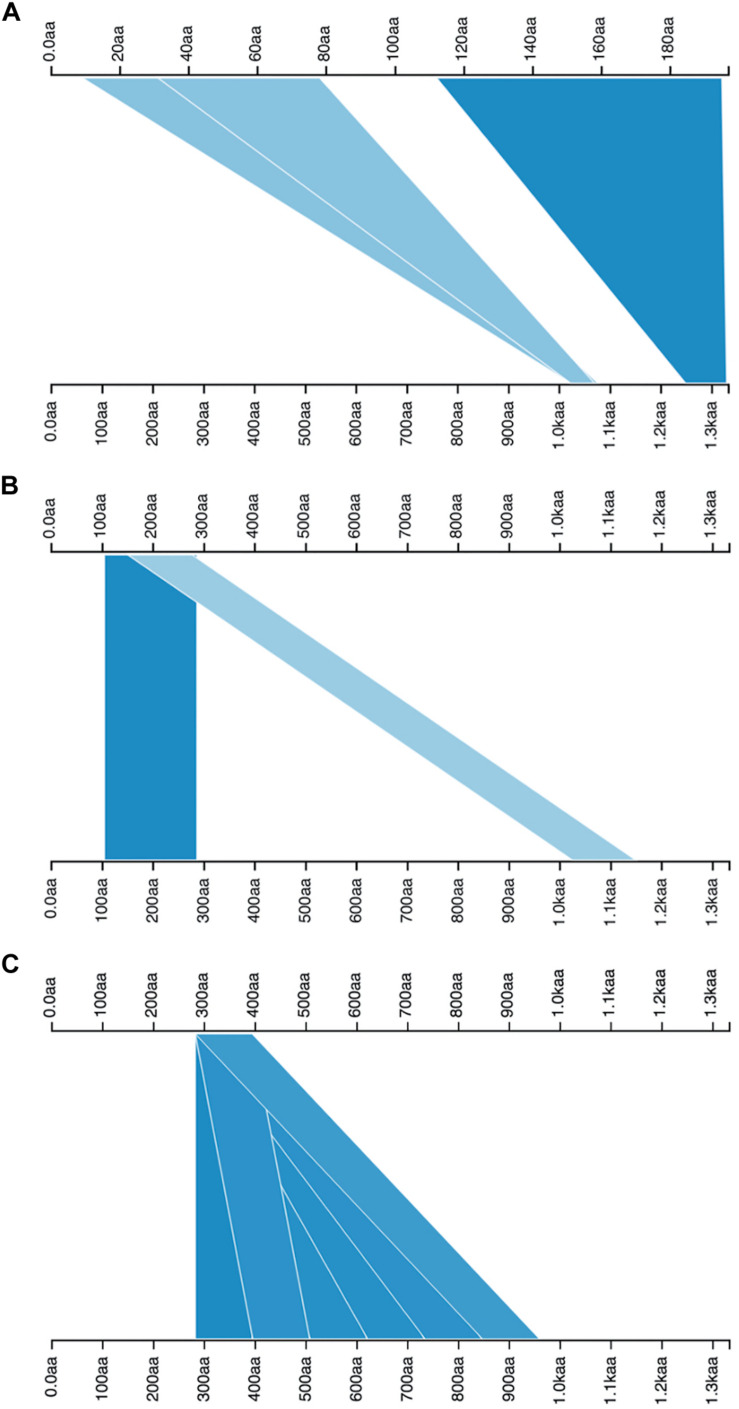
Visualization of pairwise alignments. The alignments between queries (top axes) and subjects (bottom axes) are illustrated by blue triangles or ribbons; a higher density of blue color shows a higher score. For each query subject pairing, there is more than one alignment. **(A)** the alignment of two TAAs in the proteome of *B. abortus* 2308, the top axis is representative of shorter TAA, and the bottom axis is BatA. The alignment hits include the head domains (light blue triangles) and membrane anchoring region (dark blue triangle). **(B)** Both axes are the BatA sequence, the positions of head domains are demarcated by blue ribbons. **(C)** The plot shows the presence of a repeat module, which is repeated six times along the sequence of BatA; both axes are BatA sequence. The pairwise BLAST results are visualized by Kablammo at http://kablammo.wasmuthlab.org/.

The tBLASTn results revealed that there are two genes in the genome of *B. abortus* 2308 that could encode proteins, containing the YadA-like membrane anchoring domain (YadA-anchor: Pfam: PF03895, InterPro: IPR005594): a 3999-base pair protein identified by locus tag “BAB1_0069,” “UniProtKB/TrEMBL:Q2YPR0: 1333 amino acids,” and a 591 base-pair one identified by locus tag “BAB1_1854,” “UniProtKB/TrEMBL:Q2YLM1: 197 amino acids.” The pairwise alignment of their amino acid sequences showed that similarities are limited to two portions (Figure 2A). The longer sequence (under acc. no. of Q2YPR0) is a more complex adhesin (BatA); therefore, it was the focus of this study.

### BatA Follows the General Architecture Rule of TAAs

To define the domain architecture of BatA, the location of the signal peptide was predicted by multiple servers initially.

The sequence is initiated by a signal peptide at N-terminus. The cleavage site is located after residue Ala_39_ (Supplementary Data 1 and Supplementary Figure 1). Specific BLAST search to confirm the existence of the signal peptide showed that the most significant BLAST hit belongs to BtaE signal peptide, which is a trimeric autotransporter from *B. suis* genome (Score = 75.1 bits, *E*-value = 9e-15).

**FIGURE 3:**
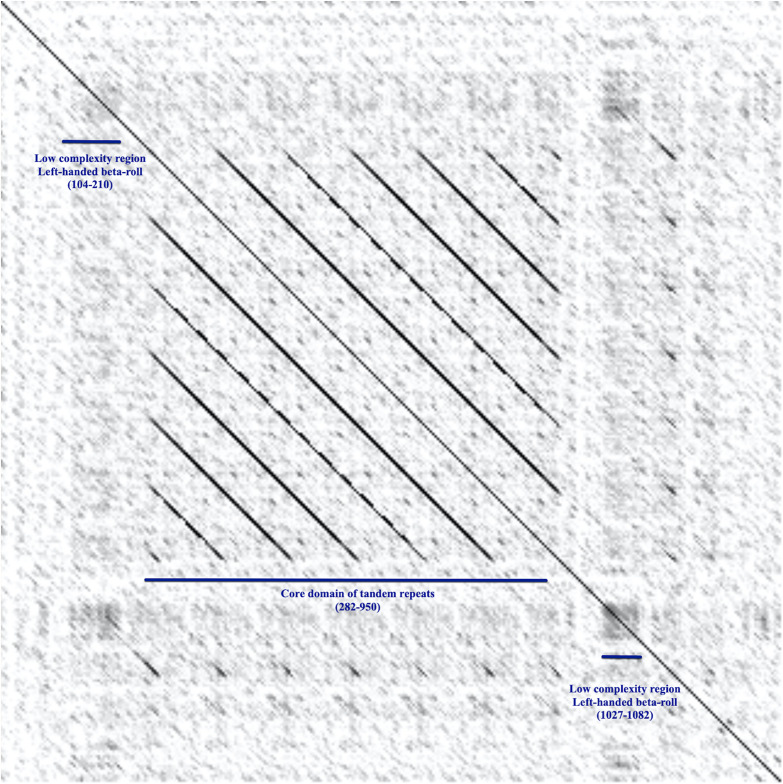
The dot-plot matrix, alignment of BatA against itself. The plot displays the alignment of the BatA amino acid sequence against itself. The horizontal and vertical axes are both the BatA amino acid sequence. The plot shows how the sequence matches to itself, therefore represents the repeat modules and low complexity regions. Each pixel corresponds to a residue in the horizontal and vertical sequences. The pixel shading represents the score of matching the residues; the darker the pixel, the higher the score. In the middle of the sequence, there is a core domain of tandem repeats, which is repeated six times within the sequence (282–950). The low complexity regions stand out as black squares.

The definition of repeat modules allowed us to perform the domain annotation of BatA as a prototype of the BTAAs. By getting clues from the dot-plot matrix results and repeat module predictors, the locations of repeated blocks were determined. The pairwise alignment function of the BALSTP tool at NCBI and visualization of the BLASTP results finalized the repeat module definition. The approach was initiated by aligning the BatA sequence with itself. The sequence contains two repetitive modules, one of which is repeated two times (Figure 2), which is a low complexity region (based on dot-plot matrix results; Figure 3), and the second one is perfectly and tandemly repeated with six repeats of the same sequence (the core domain of tandem repeats, Figures 2C, 3).

As expected, the protein ends with a potential membrane anchoring region. The transmembrane barrel is connected to the passenger by a coiled-coil domain (1208–1251) containing the period of six heptads. Short coiled-coils also mediate the connection of other segments to each other.

### Hidden Markov Model to Template the Search for Homology Modeling

Several attempts were made for collecting the proper templates for each domain block of BatA. Due to the high sequence variability of TAAs, sequence identity was not an appropriate criterion to measure the relationship. Thus, to achieve more reliable results, a Hidden Markov model profiling was utilized. A search against alignment databases such as Pfam and SMART by HHpred provides an alternative approach to identify the proper structural templates. HHpred introduces the homologous structures in a more informative manner than the BLAST search. The templates with a probability larger than 95% were selected as the final templates for homology modeling. This criterion suggests that the homology is nearly certain.

### Knowledge-Based Template Selection

The template library included some irrelevant members, suggesting that artificial intelligence was not sufficient for template selection. Therefore, a knowledge-based approach was adapted to select the templates; it means that: (i) the template should be biologically suggestive or reasonable. The organismic origin and function of the templates were also taken into account. (ii) the secondary structures of the templates should be in good agreement with those of the query. (iii) the templates should be the members of the same superfamily. This approach considerably ruled out the irrelevant hits. The existence of famous conserved motives (the result of the primary sequence analysis) was also considered (The identified patterns with their descriptions are summarized in Table 1). Taken these criteria into account, the spurious templates were filtered out, and a library of the templates that was proportional to each part of the protein was built (Table 2). In general, the alignments of templates with different parts of the protein showed a low level of sequence identity (Supplementary Data 1 and Supplementary Figure 2).

**TABLE 1:** Consensus patterns found in the primary sequence of BatA.

Pattern	Start position	Description	References
DAVN	265, 378, 491, 604, 717, 830, 943, 1137, 1204	Connects beta to alpha (reading from N-to-C)	Hartmann et al., 2012; Bassler et al., 2015
KYFHANS	1016	Alpha to a Beta connector	Hartmann et al., 2012; Bassler et al., 2015
FGG	992	A 3-stranded beta-meander inserted to coiled-coil	Szczesny and Lupas, 2008; Bassler et al., 2015
GID	356, 469, 582, 695, 808, 921	A variant of GIN	Roche et al., 2018
AXG	Multiple	The inner part of YadA like head	Bassler et al., 2015
NXYTD	1238	A right-handed coiled-coil segment to left-handed coiled-coil	Hartmann et al., 2012
S-[LVI]-[AT]-[IL]-G	1063, 1076	Forms the central structural scaffold of the left-handed parallel beta-roll	Nummelin et al., 2004; Mühlenkamp et al., 2015

**TABLE 2:** Selected templates for building tertiary structures of each block.

Position	Template name	Sequence identity	Sequence similarity	Probability	*e*-value^*b*^	Description
104–281	3pr7	14.4	24.12	98.18	3e-7	Multi-functional and mechanosensitive receptor-binding activity of the *Moraxella catarrhalis* adhesin UspA1
	1p9h	9.64	20.48	98.15	5.4e-7	Crystal structure of the collagen-binding domain of *Yersinia* adhesin YadA
	2xqh	6.67	17.33	98.14	1.7e-7	Crystal structure of an immunoglobulin-binding fragment of the trimeric autotransporter adhesin EibD
	3s6l	10.96	20.61	98.14	8.6e-7	Crystal structure of a YadA-like head domain of the trimeric autotransporter adhesin BoaA from *Burkholderia pseudomallei* solved by iodide ion SAD phasing
282–394	3d9x	39.67	53.72	97.07	0.00088	Structure of the head of the *Bartonella* adhesin BadA
	4lgo	27.42	43.55	96.5	0.01	Crystal Structure of N-terminal domain 1 of VompD from *Bartonella quintana*
950–1151	3wpa	7.69	18.62	97.56	0.0000056	*Acinetobacter* sp. Tol 5 AtaA C-terminal stalk_FL fused to GCN4 adaptors (CstalkFL)
	2yo2	10.43	22.17	97.41	0.0000096	*Salmonella enterica* SadA 255–358 fused to GCN4 adaptors
	3laa	35.98	46.26	97.4	0.000089	Crystal structure of the trimeric autotransporter adhesin head domain BpaA from *Burkholderia pseudomallei*
	3wpr	12.87	25.74	97.31	0.000024	*Acinetobacter* sp. Tol 5 AtaA N-terminal half of C-terminal stalk fused to GCN4 adaptors (CstalkN)
1152–1192	2p5z	16.67	40.48	NA	NA	The *E. coli* c3393 protein, a component of the type VI secretion system
1193–1333	3emo	24.1	37.95	99.76	1e-20	Crystal structure of transmembrane Hia 973–1098
	2gr7	28.17	44.37	99.67	3.1e-17	Hia 992–1098
	2lme	11.11	20.47	99.51	7.4e-15	Solid-state NMR structure of the membrane anchor domain of the trimeric autotransporter YadA

*1-The secondary structure similarities were taken into account when the probability was estimated. 2-The E-value is a measure of statistical significance. At E-values below one, matches are significant.*

### Structural Analysis Finalized the Domain Annotation of BatA

Since many templates were found for different parts of the protein, the homology modeling approach was exploited to build the monomeric structures with various computational methods. Multiple tertiary structures were built for each block of the protein. Amongst, the best-qualified models -the ones that matched the consensus patterns and secondary structures-were selected. The secondary structure components of BatA were predicted to be 11% helix, 27% extended strand, and 60% coil (Supplementary Data 1 and Supplementary Figure 3). All the qualified monomers were built by a modeler software.

These structures satisfied all the criteria considered to be essential for quaternary structure assembly (favored rotamers, Ramachandran favored, bonds, and angles). Hence, they were submitted to Galaxyhomomer to build their trimeric complexes. The resulting library was evaluated in the term of overall quality (Table 3). All building blocks of the protein were built (the PDB file of the modeled structures are provided as a compressed Supplementary File).

**TABLE 3:** Quality assessment of the built structures.

Protein geometry	104–286	395–507	953–1151	1152–1192	1193–1333	3ntn	3wpr	3emo	Goal
Poor rotamers	3 (0.73%)	0	0	0	0	14 (2.96%)	16 (3.25%)	39 (14.94%)	<0.3%
Favored rotamers	405 (98.54%)	264 (100%)	429 (98.62%)	105 (94.59%)	246 (95.35%)	439 (92.81%)	455 (92.48%)	179 (68.58%)	>98%
Ramachandran outliers	12 (2.22%)	0	6 (1.00%)	3 (2.44%)	12 (3.45%)	1 (0.16%)	0	5 (1.34%)	<0.05%
Ramachandran favored	468 (86.67%)	315 (94.595)	573 (95.50%)	117 (95.12%)	291 (83.62%)	596 (95.06)	638 (99.07%)	331 (88.98%)	>98%
Bad bonds:	0/3906	0/2454	0/4317	0/1011	0/2721	0/4626	0/4625	0/2623	0%
Bad angles:	51/5286 (0.96%)	48/3342 (1.44%)	66/5865 (1.13%)	6/1368 (0.44%)	45/3660 (3.62)	71/6262 (0.11)	0/6314	0/3558	<0.1%
Template	3ntn	3wpr	3wpr		3emo				

To define the domain architecture of BatA, a structural comparison was made (including superimposition and visual inspection).

The abundant domains were head, GIN, TrpRing, and coiled-coils. Details on the identified domains are provided in Supplementary Data 1. Briefly, low complexity regions were found at the N-terminus YadA like head and the C-terminus YadA like head, which are left-handed beta rolls, common in TAA architectures. Both the head domains at the N-terminus contain an inserted motif called HIM (Head Insert Motif), which extend downwards over the neck to connect the heads to a short coiled-coil region. The N-terminus head is connected to the TrpRing; and the C- terminus head is connected to a coiled-coil and FGG.

A core domain of repeated modules is an evident architecture within the sequence, which is related to the tripartite architecture of the TrpRing-GIN-Neck domain (this arrangement is appeared to be the crucial repeated modular units). This sequence is perfectly and tandemly repeated six times within the sequence of BatA. TrpRing is an interleaved head domain, parallel to the fiber axis. GIN domain is located after the TrpRing domain. GIN is a traversal head perpendicular to the fiber axis. The domain is connected to a short coiled-coil region by a neck connector.

Following the tandem repeats, there is a region composed of FGG-HANS- YLHead –HIM, which is connected to a narrower coiled-coil by a neck connector domain. FGG is a 3-stranded beta meander inserted into the coiled-coil region, and HANS is a short beta hairpin interacting with the YLHead.

At the C-terminus, the protein ends with a membrane anchoring region. The membrane channel is embedded in the membrane by a β-barrel. The membrane anchoring domain virtually represents a similar structure in all TAAs and forms a β-barrel pore containing 12 transmembrane beta strands (each subunit includes four beta strands). The BatA β-barrel has an 18.19 Angstrom-diameter central channel. The length of the barrel is estimated as 37.86 angstroms (Supplementary Data 1 and Supplementary Figure 4). The β-barrel is traversed by three N-terminal α-helices, one from each subunit.

**FIGURE 4:**
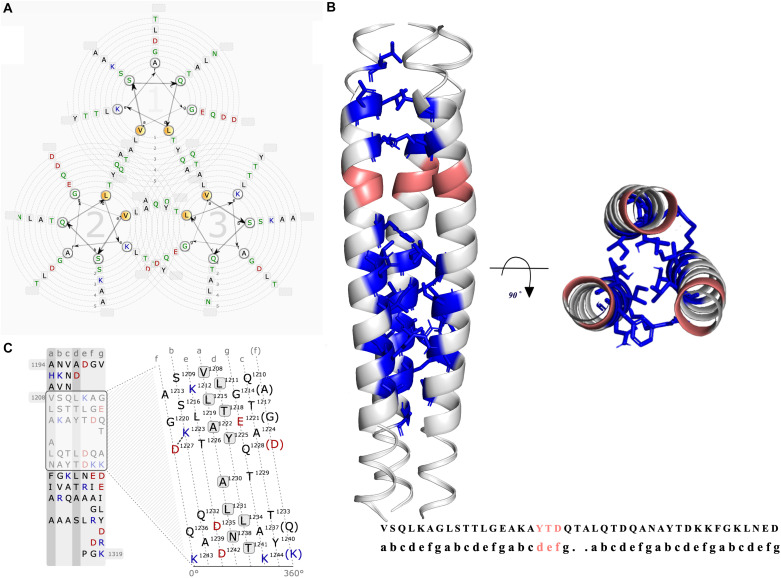
Graphical representation of the coiled-coil region of BatA. **(A)** The helical wheel view of the coiled-coil region is presented. The possible residue interactions inside a predicted coiled-coil are visualized. Each helix is represented by a wheel that is made up of seven groups (a,b,c,d,e,f,g) with the residues sorted according to the predicted heptad pattern. The sequence starts in the most inner circle of the wheel-spiral and follows in an arrow-indicated fashion to the outside. The heptad positions a and d are highlighted in orange and are oriented to the opposing wheel, representing the inner side of a coiled-coil helix. **(B)** The coiled-coil region located at the C-terminal of the protein is presented by three different colors for each chain. The location of YDD and YTD motifs (transition site of the right-handed coiled-coil segment to the left-handed coiled-coil) are colored pinkish-orange (deep salmon). The quaternary interaction of the three alpha-helices are presented with the Knob-into-holes and colored in blue. **(C)** The lower left graph is the illustration of the interaction net of an open-cut single alpha helix. In this view, one helix is cut and flattened. The flattened helix is made along the surface in the direction of the helix axis. The heptad repeat pattern, as a basis to divide the residues, is presented in strict columns, the cut is made along the column. Solid and dashed connections represent the strong (black lines) and weak (dashed gray) types of interactions between the residues.

The coiled-coils are bundles of alpha helices; typically, hydrophobic residues organized in a reiterating pattern of seven residues. The position of coiled-coils represents *abcdefg* (Figure 4). Moreover, the NXYTD motif, the site for transition of the right-handed coiled-coil to the left handed one, is present in C-terminal coiled-coil domain (Figure 4 and Table 1).

**FIGURE 5:**
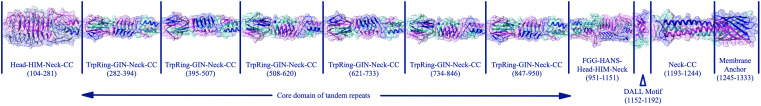
The complete assembly of the BatA structure. The complete structure of BatA is shown as cartoons with transplant surfaces. The position of each domain within the amino acid sequence of the protein is given at the bottom of each domain.

The passenger domain in this protein is composed of heads, connectors, and coiled-coils. The mature protein is the result of wrapping three identical chains around an axis to build a ∼85.4-nanometer nano-fiber (Figure 5).

### Clustering the BTAAs

**FIGURE 6:**
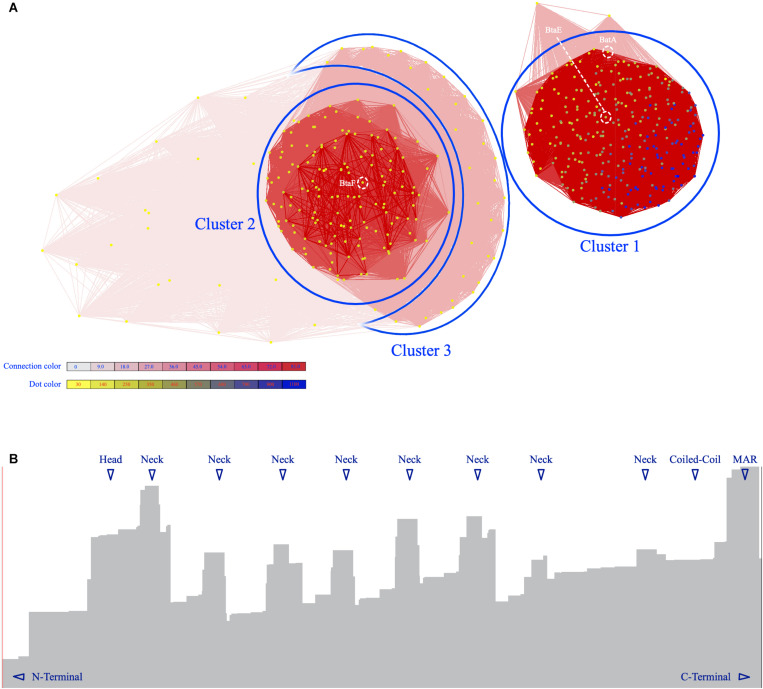
The graphical layout of 490 BTAA amino acid sequences. **(A)** The Fruchterman–Reingold graph layout algorithm to visualize pairwise sequence similarities. Sequences are represented by vertices in the graph, BLAST high scoring segment pairs (HSPs) are shown as edges connecting vertices and provide attractive forces proportional to the negative logarithm of the HSP’s *P* value (*P* value cutoff: 10^– 6^), different clusters are indicated by numbers. BatA and BtaE are members of cluster 1; BtaF in member of cluster 2. The vertices are colored based on the sequence length. The redness intensity of connecting edges is proportional to attraction forces. **(B)** The distribution of HSPs over the BatA sequence. The *X*-axis is the amino acid sequence and the *Y*-axis is the level of distribution. The highest levels are indicated by triangles.

To gain a better insight into the characteristics of TAAs in the *Brucellaceae* family, an all-against-all approach was employed to cluster the BTAA amino acid sequences. Based on all-against-all pairwise similarities, TAAs were clustered into three distinct and clear clusters and few scattered sequences (Figure 6A). The amino acid length of sequences ranged from 86 (locus tag: BSPT1_II1279, *B. suis* bv. 2 strain PT09143) to 1559 (locus tag: P050_01136, *B. abortus* 90-12178). The first cluster included the longest sequences and involved all species except for *B. pinnipedialis.* A 1333 amino acid sequence was the most abundant sequence in the population (see Supplementary Data 2, CLANS tab, for details of different clusters). In general, the sequence lengths of BTAAs are heterogeneous [compare it with *Acinetobacter*, which is highly heterogenous (Rahbar et al., 2019)]. BatA is a member of the cluster 1. Moreover, two well-defined BTAAs, viz. BtaE and BtaF, are members of the cluster 1 and the cluster 2, respectively.

### Topology of BatA

**FIGURE 7:**
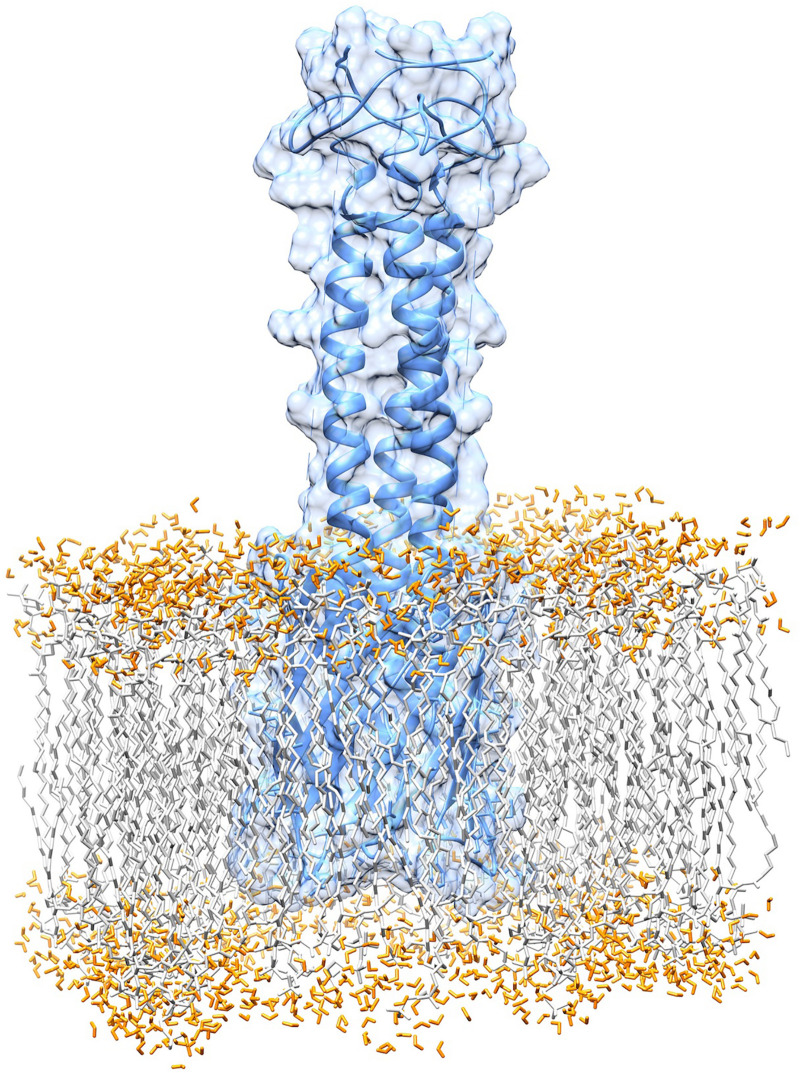
Graphical representation of the membrane anchoring domain of BatA. The alignment of the membrane anchoring region with the lipid bilayer of the membrane is provided.

The results of topology prediction indicated that the BatA protein is an outer membrane beta-barrel protein. The position for transmembrane strands (TM) was predicted to be: TM1: 70–79, TM2: 83–92, TM3: 97–106, and TM4: 110–120 by BOCTOPUS, TM1: 68–78, TM2: 97–105, and TM3: 110–120 by PRED TMBB and TM1: 52–59, TM2: 71–79, TM3: 84–92, and TM4: 112–122 by PRED TMBB2. The predictions of the BOCTOPUS and PRED TMBB2 are highly concordant in regards to the number of transmembrane β-strands, however, the PRED TMBB predicted an outer membrane strand spanning the amino acids 79–96. The globular beta barrel-shaped domain of the molecule I is located within the membrane, while the helical stems of the molecule are sprouted out of the membrane. Collectively, the topology predictions and membrane alignment studies could be considered as a reliable source for the prediction BatA structure. Figure 7 shows the BatA structure is embedded within the lipid membrane in the presence of water molecules.

### Flexibility of Domains

**FIGURE 8:**
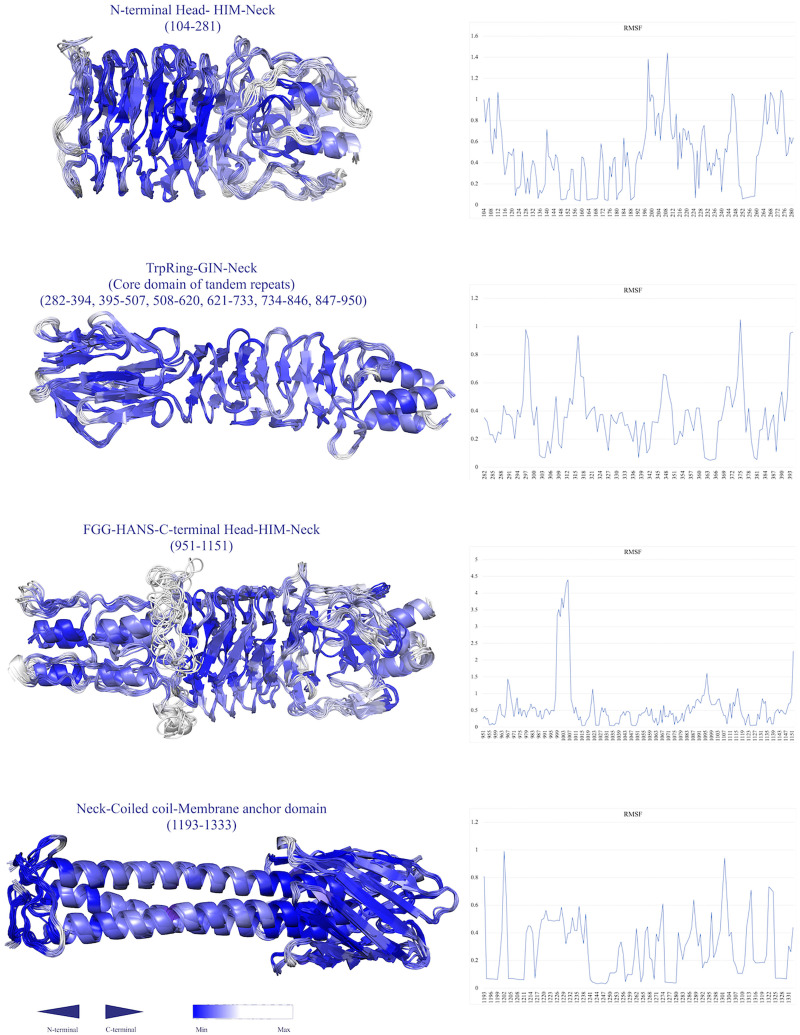
Simulations of the conformational flexibility of the structures by a Monte Carlo-based method (CABS dynamics). The residue fluctuation profiles are shown on the right and the ensemble fluctuation models are displayed on the left. The structures are labeled by their name and position. The residues are colored by a gradient of blue color (the higher the RMSF, the lower the density of blue color.

To estimate the resiliency and toughness of the structures, a coarse-grained dynamics approach was employed, at which the high root mean square fluctuation (RMSF) value is indicative of high residue flexibility. The highest flexibility was associated with the HANS, HIM, and neck connectors, respectively, (Figure 8). Conversely, the beta hairpins represented lower RMSF values. The residues of YLHead domains and the membrane barrel had far lower RMSF values, representing their less flexibility.

### Large Interior Cavities of the Coiled-Coil Region

**FIGURE 9:**
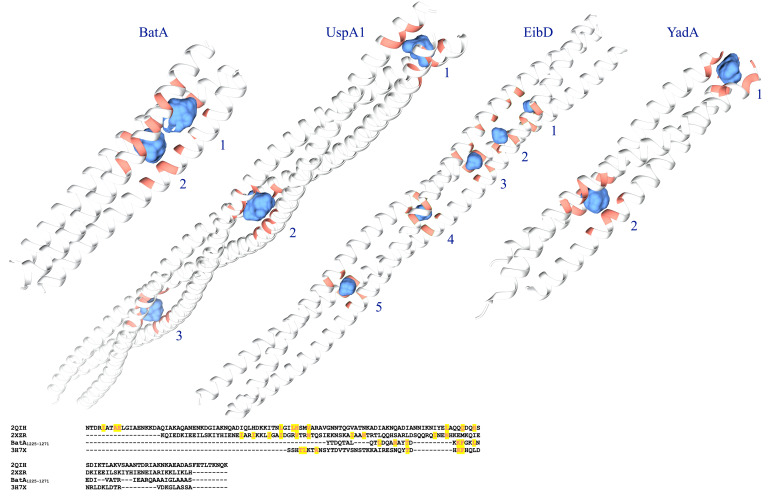
Graphical visualization of the major cavities. The cavities within the coiled-coil structure of BatA and the three well defined TAA coiled-coil structures are presented by blue surfaces. The structures are labeled by protein names; cavities are labeled by the Arabic numbers; details of the volumes are provided in Table 5. The lower panel shows the sequence alignment of these coiled-coil structures; the residues that surround the cavities are highlighted.

To examine the possible effect of cavities within the coiled-coil region of BatA, the existence and volume of cavities were assessed and measured. The largest cavities measured by the two servers (see “Materials and Methods” section), were presented in Figure 9. The numerical data representing the volume and surface of cavities are summarized in Table 4. As is evident in the table, the cavities of BatA coiled-coil are comparable to the other three examined molecules in term of volume. Moreover, the surrounding residues are similar to the conserved coiled-coil segment of TAA of *Y. enterocolitica*. The second large cavity in the coiled-coil structure of BatA is located in the transition site of this section (right-handed coiled-coil to left-handed coiled-coil; also see Figure 4).

**TABLE 4:** The volume and surface area of the interior coiled-coil cavities of four TAAs.

Description	Pocket number	MS volume*	Pocket MS area**
BatA	1	181.9	174.4
	2	178.3	164.6
2QIH	1	114	137.7
	2	235.9	219.9
	3	176.6	181.4
2XZR	1	34.1	52.1
	2	56.1	79.3
	3	60.3	78.2
	4	63.3	80.7
	5	64.8	82.2
3H7X	1	128.3	143.4
	2	138.3	138

*Pocket volume based on the molecular surface. **Pocket molecular surface area.

### Trimeric Autotransporters Are Present in all Members of *Brucellaceae*

To determine the distribution of TAA encoding genes in *Brucellaceae*, a tBLASTn search against all genomes of the family members was conducted by querying the consensus amino acid sequence of the membrane anchoring region of TAAs. All significant BLAST hits (*E*-value < 10^–6^) were mapped to the phylogenetic tree of *Brucellaceae*. The results showed that the TAA encoding genes were present in all 300 leaves of the tree; suggesting that all species share a common ancestor (Supplementary Data 1 and Supplementary Figure 4). Moreover, 201 leaves contained two TAA encoding genes (orange leaves in Supplementary Figure 4).

### Evolutionary Analysis

#### The Nucleotide Substitution Pattern Is Homogeneous in the Dataset

For estimating the best evolutionary model, the dataset involving 490 nucleotide sequences was analyzed by the model selection function of MEGA 7. Codon positions included were 1st + 2nd + 3rd + Non-coding. The positions containing gaps and missing data were eliminated. The best model for a proper description of the substitution pattern of nucleotide collection of BTAA membrane anchoring region (it is the model with the lowest Bayesian information criterion; BIC), was Kimura 2 parameter (Kimura, 1980; BIC: 12063.54 versus higher values of other models, Supplementary Data 2).

To examine whether the pattern of nucleotide substitution was homogenous, the disparity index tests of substitution patterns were performed for each pair of BTAA nucleotide sequences. In the disparity index (ID) test using all (1st + 2nd + 3rd) codons at the 5% significance level, the null hypothesis of neutrality could not be rejected for membrane anchoring region (Supplementary Data 2). Therefore, all sequences were retained for further analysis.

#### Codon Usage and G + C Contents

No significant bias was observed in the codon usage of the BTAA encoding genes. The mean differences between adaptiveness values ranged from 20.45% to 35.88%. The highest value attributed to the TAA genes of *B. pinnipedialis* (versus that of Omp31: 28.14%; Supplementary Data 1 and Supplementary Figure 5). The G + C content of TAA encoding genes was ∼54% (versus G + C content of *Brucellaceae*: 57.2%; Sankarasubramanian et al., 2016b).

#### Gene Duplication

To trace possible duplication events, an unrooted gene tree was built based on the nucleotide sequences of the membrane anchoring region. The search for duplication events was performed by finding the placement of the root of branches that produced the minimum number of duplication events. There were at least 14 gene duplication events within the tree (data not shown).

#### Recombination

To define the recombination events, the whole sequences of BTAA encoding genes were analyzed by GARD. Substantial evidence for recombination breakpoints was found. The alignment contained 4691 potential breakpoints, translating into a search space of 11005086 models with up to two breakpoints, of which, 0.01% was explored by the genetic algorithm. The AICc [Akaike Information Criterion (AIC; Sugiura, 1978)] score allows for different topologies between segments (26929.4), the model then assumes a similar number of trees for all the partitions inferred by GARD, but allows for different branch lengths between partitions (28750.0). Comparing the AICc score of the best fitting GARD model suggested that the multiple tree model is preferred over the single tree model (based on an evidence ratio of greater than 100). At least one of the breakpoints reflects a true topological incongruence.

#### Distances and Diversities

In order to measure the diversity and distances within BTAAs, all nucleotide sequences of the membrane anchoring region of BTAAs were grouped by their related species. The highest divergence was found between *B. pinnipedialis* and other species (Table 5). The number of base substitutions per site was calculated by averaging the substitution rate over all the sequence pairs within groups (Table 5). Analyses were conducted using the Kimura 2-parameter model. The included codon positions were 1st + 2nd + 3rd + Non-coding. All positions containing gaps and missing data were eliminated.

**TABLE 5:** Estimates of evolutionary divergence over sequence pairs between groups.

Column1	*B. melitensis* olumn2	*B. abortus*3	*B. suis*n4	*Brucella_sp* 5	*B. canis*	*B. ovis*	*B. pinnipedialis*	*B. ceti* n9	*B. neotomae* 0
*B. melitensis*									
*B. abortus*	0.5								
*B. suis*	0.5	0.48							
*Brucella_sp.*	0.42	0.46	0.45						
*B. canis*	0.37	0.44	0.41	0.22					
*B. ovis*	0.66	0.67	0.7	0.65	0.77				
*B. pinnipedialis*	0.79	0.58	0.55	0.76	1.01	0.86			
*B. ceti*	0.43	0.46	0.46	0.39	0.29	0.64	0.71		
*B. neotomae*	0.52	0.49	0.48	0.49	0.48	0.68	0.51	0.48	
*B. microti*	0.52	0.49	0.48	0.49	0.48	0.68	0.51	0.48	0.48

*The number of base substitutions per site from averaging over all the sequence pairs between groups are displayed.*

### No Evidence of Positive or Purifying Selections

The *Z*-test for codon-based selection showed no evidence of positive or purifying selection in BTAA encoding genes (Supplementary Data 2), so the null hypothesis of neutral selection could not be rejected in favor of the alternative hypotheses of positive and purifying selection. Moreover, Fisher’s exact test of neutrality was not significant in rejecting the neutral selection hypothesis in favor of positive selection (Supplementary Data 2). These data suggest that mutations are neutral rather than beneficial.

### Exploring the BTAAs

This section contemplates the overall architecture of BTAAs. The batch analysis of all sequences revealed that the Sec signal peptide is predicted to exist in 17% of the sequences; 48% contain Tat signal peptide, and the remaining sequences had no detectable signal peptides. Interestingly, no correlation was observed between sequence length and the existence of signal peptides. The lengthiest sequences belong to *B. abortus* and *B. melitensis* (Supplementary Data 1 and Supplementary Table 1). As is summarized in Supplementary Table 1, the presence of identical sequences is common in the *Brucella* genus, consistent with the high level of genome identity in this genus.

**FIGURE 10:**
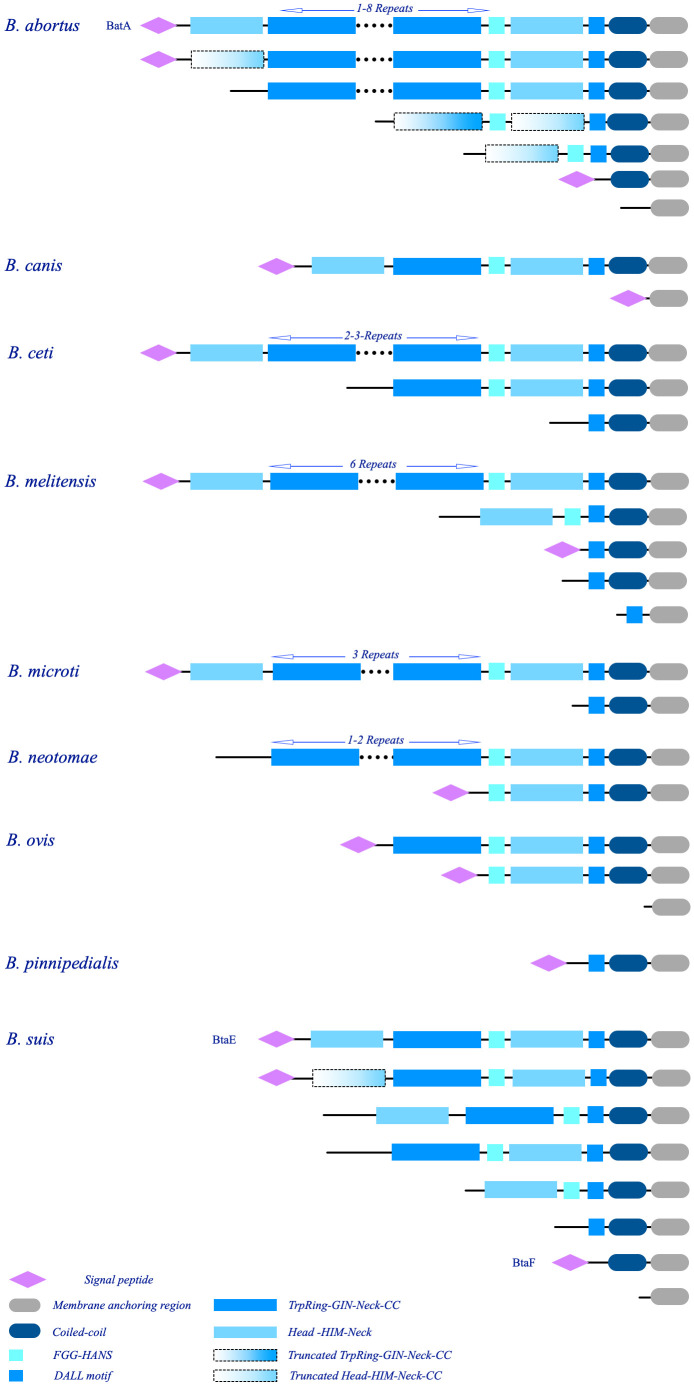
Schematic illustration of the major architectures of BTAAs. The observed architectures of BTAAs are presented; each group is labeled by the hosted species. The number of the repeat modules are shown at the top of the respective structures. The architectures of BatA, BtaE, and BtaF are assigned by protein names; notably, similar architectures to these three proteins can be found in the library (Supplementary Data 2). The schematic domains are identified at the bottom.

The sequences are rich in coiled-coil regions. Moreover, 55% of the dataset contains repeat modules. The repeat modules include a tripartite module of TrpRing-GIN-Neck, which is repeated multiple times in many sequences, such as the BTAAs from *B. abortus, B. melitensis, B. ceti, B. microti*, and *B. neotomae* genomes, or is present in one copy in the TAAs of *B. canis, B. ovis, B. suis*, or is completely absent in the TAAs of *B. pinnipedialis*. The other common repeat modules are short sequences within the head domains. The head domains followed by HIM and Neck connectors are present in both the N-terminal and C-terminal regions of many BTAAs. Moreover, truncated heads and TrpRings can be observed in BTAA architectures (Figure 10).

This paper defined the BatA as a prototypical TAA in the *Brucella* genomes. The architecture of BatA is a combination of all observed domains and motifs; however, although the exact architecture is not presented in all genomes (except for *B. melitensis*), similar architecture is presented in other species (except for *B. pinnipedialis*). As is evident in Figure 10, truncated heads and TrpRings are obvious in *B. abortus* and *B. suis*. Further, small TAAs including a single membrane anchoring region is presented in all species (except for *B. suis*).

The architectures of the two previously defined BTAAs, BtaE, and BtaF, are as follows: BtaF: Signal-peptide-N-terminal head-TrpRing-GIN-Neck-FGG-C-terminal Head-DALL-Coiled-coil-Membrane anchoring region; BtaE: Signal-Peptide-extended coiled-coil-Membrane anchoring region. The relatively small sequences of approximately 200 amino acid long, contain extended coiled-coils. Details of BTAAs are provided in Supplementary Data 2.

Duplicated genes are further apart in one chromosome and often in both forward and reverse strands. Therefore, both orthologous, and paralogs TAA genes are present in the *Brucella* genomes. The details of BTAA are provided in Supplementary Data 2 and Figure 10.

## Discussion

The present study was aimed at identifying and characterizing the TAAs in Brucellae via *in silico* approach. Computational tools provide a means to attain structural data (Negahdaripour et al., 2016) which is, by itself, a gateway to access a massive plethora of biological information (Negahdaripour et al., 2017). End conclusions are context-dependent, yet are generally derived from the broad concept of homology.

It appears that the few domains shared by the whole *Brucellae* population are responsible for the observed heterogeneity in the sequence length of BTAAs. Significant differences were also found in the arrangement and repeat frequencies of TAA domains in *Brucella* species. Paralogs of different sequence lengths are common within the species, which is indicative of duplication events; this conclusion is also supported by the evolutionary analyzes (see the evolutionary analysis). However, in comparison with Gammaproteobacterial TAAs, such as *Acinetobacter* species TAAs (Rahbar et al., 2019), the length of the sequences is shorter and less heterogeneous.

Moreover, the distribution of BLAST HSPs across the sequences revealed the existence of a few structural building blocks in the entire population (Figure 6B). Differences in the structural blocks of BTAAs are limited to the repeat frequencies of these blocks. These differences may play a variety of roles in host preferences and tissue tropism. This hypothesis was previously suggested by Chain et al. (2005) and Ruiz-Ranwez et al. (2013b) as well. The result of the disparity index test supported the homogeneity of membrane anchoring sequences, suggesting that TAA genes have evolved via the same or similar evolutionary processes. Moreover, the presence of the TAA-encoding genes in all members of the family implies that these genes share a common ancestor. This conclusion is further supported by a lack of evidence for conjugation (no plasmid is identified in *Brucellaceae*, except for the Ochrobacteria, which harbors TAA encoding plasmids), as well as similar G + C contents and the paucity of codon biases.

The differences in sequence length, domain complexity, and the existence of paralogs could be explained by recombination and duplication events. It can be deduced that all sequences are derived from a common ancestor, which can, in turn, provide insight into the existence of population-wise domains. The observed heterogeneity could be the consequence of genetic events such as the duplication and recombination events that occurred intraspecies. This conclusion is consistent with the evolutionary history of *Brucellaceae*.

According to a previous genomic comparison study, 95% DNA identity was revealed across the genomes of *B. abortus, B melitensis, B. canis, B. neotomae*, and *B. ovis*, which led to the assumption that they have all diverged from a common ancestor that was most likely very close to *B. ovis* (Foster et al., 2009).

Introducing a prototype sequence is commonly used to define a protein family (Andreeva et al., 2013). This approach is more pronounced for TAAs, which share common structural and functional properties (Linke et al., 2006). As is the case here, it seems rational to analyze a prototype sequence and make comparisons to define the entire members of the gene family.

It has been shown that due to the TAA sequence diversity, alignment and tree-based analyzes are not suitable to investigate the properties of TAAs (Pina et al., 2009; Fialho and Mil-Homens, 2011). Hence, all-against-all BLAST was harnessed here, as an appropriate alternative to handle the massive data, including very disparate sequences (Rahbar et al., 2019). Furthermore, it is difficult to annotate the domains of TAAs due to their considerable sequence diversity, the various patterns of domain arrangements, and the limited coverage of sequences by known structures (Meng et al., 2006; Bassler et al., 2015). This was evident in our study following the alignment and observation of the low level of sequence similarity. Due to the same reasons, general domain annotators, such as Pfam, perform poorly in recognizing the TAA domains (Szczesny and Lupas, 2008). Given these circumstances, invoking an innovative and integrated approach could be of value. The solution could be an structural analysis, especially when the homologous structures are available via the process of homology modeling (Montgomerie et al., 2008; Steinegger et al., 2019; Zheng et al., 2019).

*Brucella abortus* is the main causative agent of brucellosis in cattle leading to abortion, infertility, and decreased milk production (Neta et al., 2010). *Brucella abortus* 2308, a highly virulent strain isolated from an aborted fetus of a cow (Jones et al., 1965), is wildly used as a reference strain in many brucellosis research (Suárez-Esquivel et al., 2016). A 1559 amino acid long TAA (locus tag: P050_01136, *B. abortus* 90–12178) from this bacteria was the largest TAA found in our dataset. The difference between BatA and the latter (P050_01136) is limited to the frequency of core repeat modules. Among the architectures defined for various TAAs, BatA has a relatively low level of complexity and the protein architecture matched to 104 similar protein architectures in the Interpro database.

Homology modeling based on the known homologous structures is the most successful approach for protein structure prediction. The phenomenon is rooted in the fact that structures diverge much more slowly than sequences, even when their sequences have diverged out of a distinguishing range (Kinch and Grishin, 2002). Sensitivity is crucial for success in homology detection, since many proteins have only remote relatives in the structure database (Söding et al., 2005). TAAs do not share global sequence similarities, although they perform common functions (Linke et al., 2006). Despite the differences in their sequences, structural similarities highlight the important role of structure in these proteins. On the other hand, it implies that various evolutionary events have manipulated the primary sequence and domain arrangements of these proteins.

By searching the databases with HHpred, the best templates were selected to generate tertiary structures. Comparing the built structures with that of the resolved structures allowed us to annotate the domain architecture of the protein.

The null hypothesis of homogeneity of N-terminal of BTAAs was rejected (data not shown); revealing a diversity in this region (the 160 nucleotides of N-terminal of BTAAs is considered to be the coding region the signal peptide).

The signal peptide in TAAs generally delivers the unfolded proteins to the Sec secretion machinery (Navarro-Garcia et al., 2004). However, in our dataset, inclusion of the N-terminus region of BTAA encoding genes resulted in a significant level of diversity. Both Tat and Sec signal peptides were predicted in the population, though many sequences did not comprise any detectable signal peptide. Therefore, while the secretion of some BTAAs is under debate, the microarray data have shown that both TAAs from the *B. abortus* 2308 genome are expressed (Kleinman et al., 2017). Although based on the prediction of interaction among secretion systems in the *Brucella* genus (Sankarasubramanian et al., 2016a), both Sec and Tat pathway proteins interact with T4SS and T5SS proteins, and it seems unlikely that Tat pathway participates in BTAAs translocation. This notion could be rooted in two facts: (i) Sec signal peptide is the most common signal peptide in TAAs in different genera; and (ii) TAAs are known to be secreted in unfolded forms (Navarro-Garcia et al., 2004; Cotter et al., 2005; Linke et al., 2006; Łyskowski et al., 2011), which is not consistent with the Tat system (Natale et al., 2008). Thus, the predicted Tat signal could probably be a computational error due to the high similarity between the targeting signals (Natale et al., 2008).

A trimeric lipoprotein in Enterobacteria is involved in the biogenesis of TAA (Grin et al., 2014) and arranged as an operon with TAAs in Enterobacteria. A similar operon has been also introduced in *Acinetobacter* Tol5 (AtaA and TpgA; Ishikawa et al., 2016). The BatA encoding gene and its downstream gene (IalB, 182 aa) are stated to constitute a bicistronic unit (Kleinman et al., 2017). IalB was predicted to be localized in the periplasmic region or the inner-membrane of *Brucellae* (in contrast to the trimeric lipoprotein of Enterobacteria and TpgA, which are outer membrane proteins). IalB has been also introduced in *Bartonella bacilliformis* as a major virulence factor with a direct role in human erythrocyte parasitism (Coleman and Minnick, 2001). The structural alignments of IalB and TpgA (TCoffee expresso package (Armougom et al., 2006); the server combines the structural information with sequence data to build alignment; the alignment is built on sequences) showed some levels of similarity (data not shown). However, our method did not provide sufficient data to interpret the relationship between BatA and IalB. Kleinman et al. (2017) proposed that this bicistronic unit is under the regulatory control of VjbR. A combinatorial control system following the association of HutC and MdrA regulatory proteins was also observed (Sieira et al., 2017). The MucR was reported to be another regulatory element for this open reading frame (Caswell et al., 2013). It seems that operon configuration is not the most likely explanation for this genomic proximity between BTAAs and IalB encoding genes. The exact relationship of these two neighbor genes (if any) is remained to be explored.

Residue fluctuation profile of the BatA domains suggests that coils were generally responsible for the highest RMSF values. The most flexible regions with the highest RMSF (coil-containing structures including HANS, HIM, and neck connectors) provide the bending sites for the nano-fiber. This property was previously noted for the HANS motif (Hartmann et al., 2012). The C-terminal coiled-coil region exhibited lower levels of flexibility, which is consistent with the fact that coiled-coils stabilize the structure of the protein (Meng et al., 2006). However, as previously ascribed to some TAAs, such as the UspA1 protein from *Moraxella catarrhalis* (Conners et al., 2008), the coiled-coil region of the *E. coli* EibD (Leo et al., 2011); and a conserved coiled-coil segment of TAA of *Y. enterocolitica* (Alvarez et al., 2010), interior cavities are the regions of deformation to allow bending the stalk. Attribution of such a role to the cavities within the C-terminal coiled-coils is not ruled out. Because, cavities of the similar volume comparable to the aforesaid proteins were observed in BTAAs.

Globular heads and beta rolls also represented a low RMSF value implying the rigidity of these domains. While both the aforesaid domains contain structurally repetitive units, it seems that repetitive structures [mostly cross beta-prisms (Roche et al., 2018)] may confer an overall toughness to the protein. This is obvious in the membrane anchoring region, which is composed of repetitive up and down beta hair-pins structures (Roche et al., 2018). These strands were in a lower RMSF state.

The primary requirement for such an adhesin to deliver its functions is its structural length. It should be long enough to pass through the surface elements of the bacterial cell and reach the receptors on the host cell. Moreover, it has to be flexible to form multiple binding sites and ensure a high affinity. The core domain of repeat modules (consisted of TrpRing), which is suspected to bind to fibronectin (Meng et al., 2008; Szczesny et al., 2008; Qin et al., 2016), along with the proper flexibility of the specific domains, provide opportunities for the adhesin to bind from multiple sites (six Trp-ring-GIN strung together by neck connectors). Thus, it could be suggested that despite the low affinity of TrpRing to fibronectin, binding from multiple sites might enhance the overall affinity and enable the protein to overcome the mechanical forces. Given the approximate length of 85 nm (BatA), it can pass through the surface components of the bacterial cells. Moreover, trimerization provides three identical faces that expose multivalent binding sites upon adhesion (Cotter et al., 2006).

It has been shown that trimerization is essential for the full functionality of TAAs (Cotter et al., 2006; Schütz et al., 2010). The main structural reason for such trimerization is hydrophobic interactions especially head domain, whose cores are extremely hydrophobic (Nummelin et al., 2004). Furthermore, the collocation of three alpha-helical coils makes a hydrophilic region (due to polar side chains) that sequesters the ions into the coiled-coils (Hartmann et al., 2009). C-terminal coiled-coils are available in almost all BTAAs. Therefore, it can be speculated that all BTAAs have minimal requirements for trimerization. However, lacking functional domains (perpendicular and interleaved heads) in many members would cast doubt on the extent of functionality of all BTAAs.

It has been proved that many TAAs are involved in biofilm formation. *Brucellae* has long been considered as a facultative intracellular pathogen in most references. However, they were re-designated as a facultative extracellular-intracellular pathogen due to their evolutionary relationship to other alpha-proteobacteria (El-Sayed and Awad, 2018). The biofilm lifestyle of *Brucella* has already been confirmed (Uzureau et al., 2007; Godefroid et al., 2010; Almirón et al., 2013). Therefore, the possible relationship of TAA with the biofilm lifestyle of *Brucellae* could be the objective of future studies.

Contrary to other genera such as *Acinetobacter*, *Burkholderia*, and *Haemophilus*, the absence of any plasmid or phage in the *Brucella* genus has ruled out the possibility for transfer of TAA genes by conjugation.

*Brucellae* coevolved with animals in a pathogenic manner. Given their broad mammalian host range, *Brucella* species are faced with different environmental conditions (phagocytic and non-phagocytic cells such as fibroblasts and epithelial cells) and consequently various stressful conditions. However, the genetic diversity in the species is relatively low. The bacterium required several components to survive within a mammalian host cell. Here, it seems that TAA is not a critical factor for the bacterial survival. The mechanism for the expression of most genes is debatable due to the deletion of relative signal peptides. Moreover, the functionality of several members is also questionable due to the lack of functional domains (i.e., various sorts of head domains). The relative abundance of synonymous and non-synonymous substitutions that have occurred in the gene sequences has been compared in the present study. The data implies that substitutions are neutral. TAA harboring phenotype is not an optimum phenotype for survival. Therefore, TAA encoding genes are not under positive selection. On the other hand, the virulence of pathogenic species was weakened by mutations in the TAA gene. These mutations did not end with the complete abolishment of virulence, which indicates that these sorts of adhesins are not critical to establish the infection. It can be assumed that the adhesion to the host cells takes place by other components. Thus, positive or purifying selection does not affect the TAA genes in *Brucellaceae*. Presumably, due to neutral mutations in the population, the non-adaptive evolution would be a well-fitted theory. Since the model does not invoke a positive selection as the driving force of fixation, the BTAA encoding genes may have evolved through a non-adaptive evolution.

## Conclusion

*Brucella* species, as zoonotic intracellular pathogens, harness an arsenal of membrane components to penetrate the host cells. The trimeric autotransporters are among the many virulence factors of *Brucellaceae*, which have evolved through a non-adaptive evolution process. The evolutionary events, sequence diversity, and structural complexity are dictated partly by the repetitive nature of these adhesins. The modifications include alterations within the repeat frequency of a few structural blocks of a common ancestral gene. The events have occurred among species without the interference of foreign sequences. The issue is traceable by computational tools. The *in silico* approach used here holds the potential for handling such sets of disparate sequences to investigate the great regularity of the living systems, especially at a molecular level. However, the proposed model herein does not rule out the existence of any other evolutionary event. Thus, other possible processes could also be identified through further studies.

## Data Availability Statement

The original contributions presented in the study are included in the article/Supplementary Material, further inquiries can be directed to the corresponding author/s.

## Author Contributions

MR proposed and designed the idea and the study, contributed to the writing of the manuscript, and discussed the results. MZ, AJ, and SK collected, processed, and analyzed the data, and were involved in the study design. NN, MN, and YF contributed to the writing of the manuscript and revised the final version. MN also edited the final version. AS contributed to the writing of the manuscript and discussed the results. YG proposed and designed the idea and the study, provided the facilities, funding, commented, and revised the manuscript. All authors contributed to the article and approved the submitted version.

## Conflict of Interest

The authors declare that the research was conducted in the absence of any commercial or financial relationships that could be construed as a potential conflict of interest.
