# Supplementary material for: Non-adaptive Evolution of Trimeric Autotransporters in Brucellaceae
Source: Front Microbiol. 2020 Nov 12;11:560667. doi: 10.3389/fmicb.2020.560667 (PMC7688925; doi:10.3389/fmicb.2020.560667)
Supplement: Supplementary file 1 [file Data_Sheet_1.docx]

*
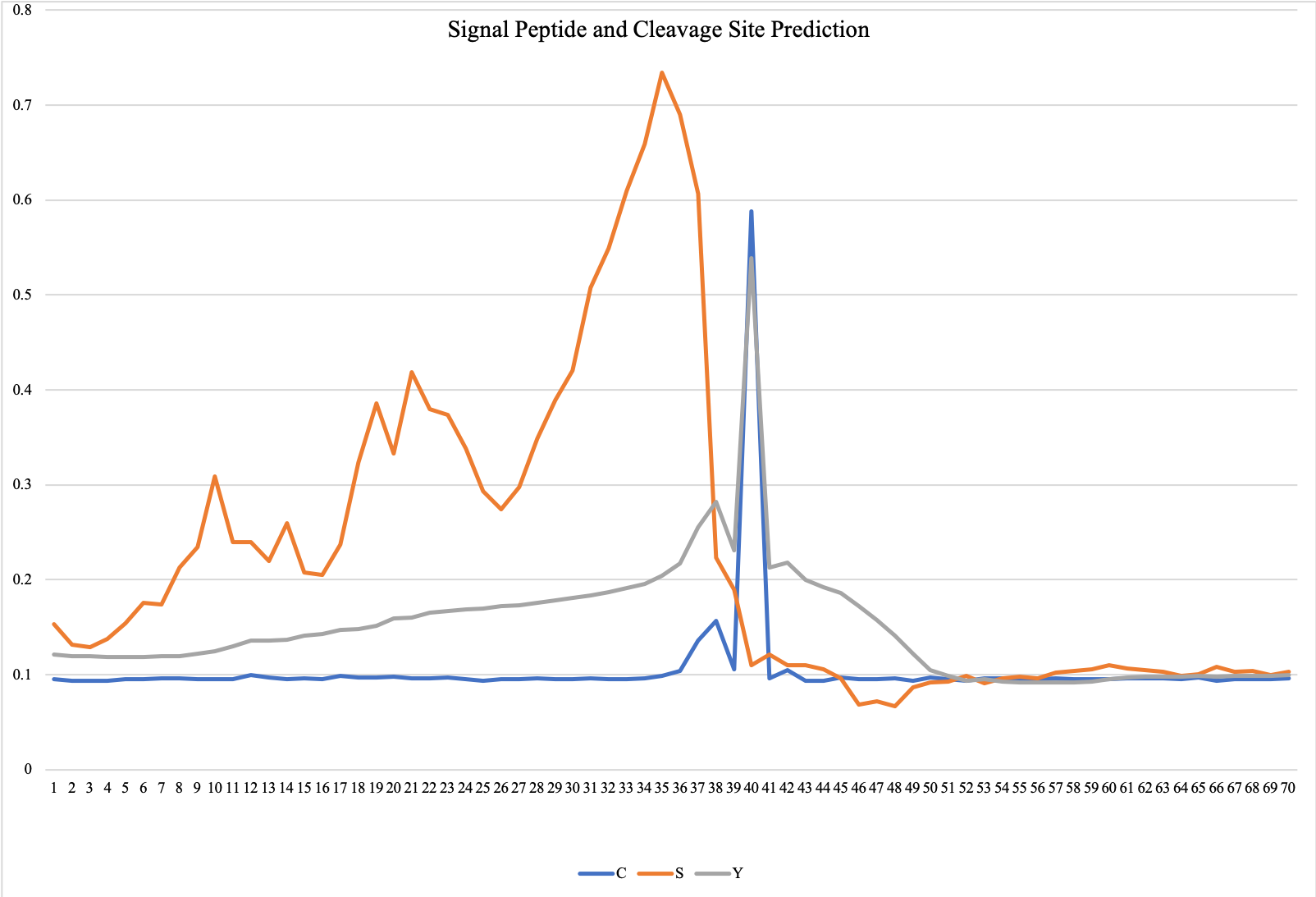
*

**Figure S1.** Signal peptide and cleavage site of BatA. The predicted cleavage site of signal peptide region based on neural networks; three series of scores include raw cleavage site (C-score) which discriminates the signal peptide cleavage site: the peak on amino acid Ala39 shows the first residue in the mature protein. Signal peptide score (S-score) represents the position of the signal peptide, and combined cleavage site score (Y-score), geometric average of the C-score and slope of S-score; the Y-score shows the precise cleavage site by distinguishing the highest C-score exactly where the S-score slope is steeped.


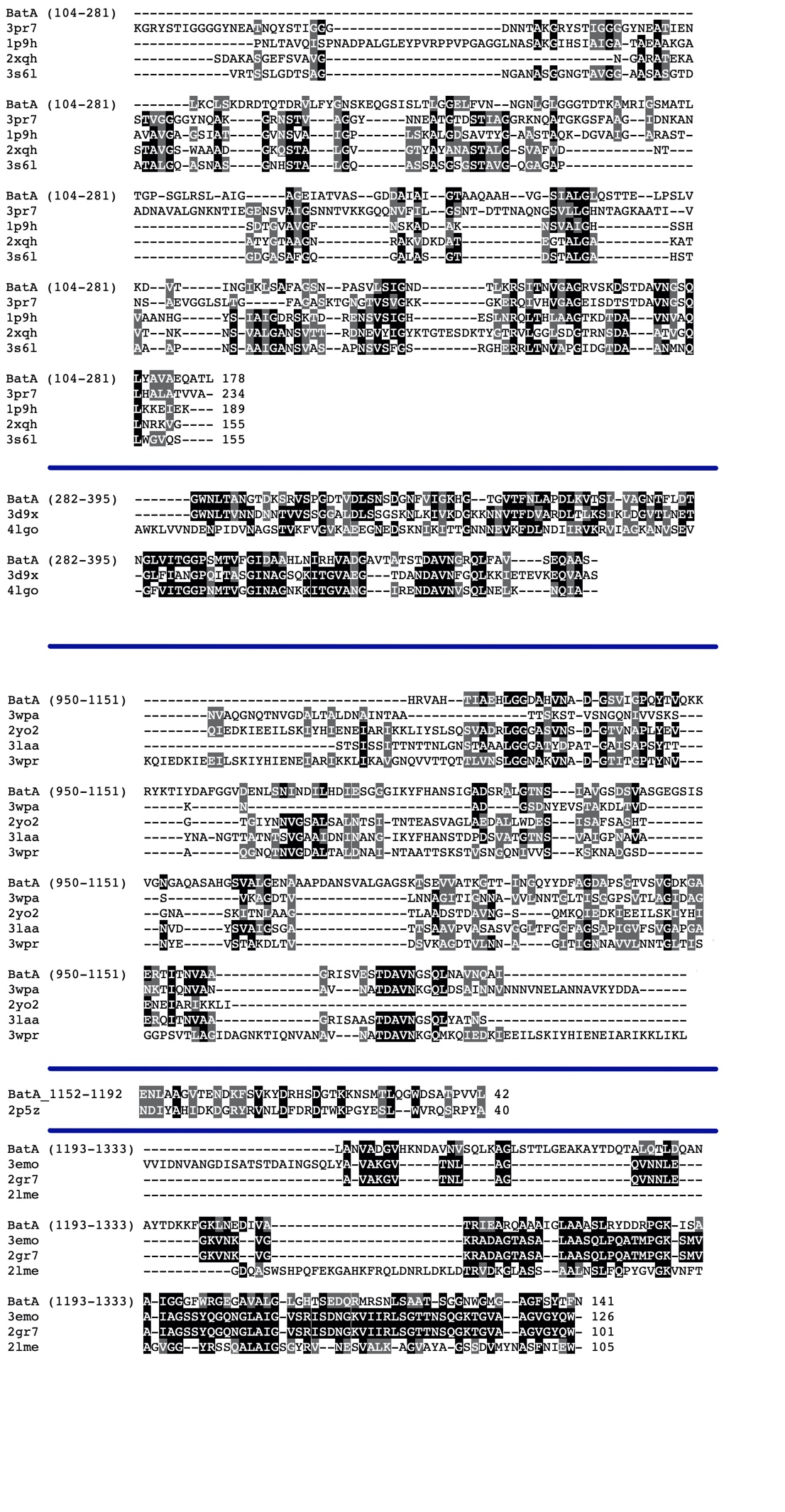


**Figure S2.** Alignments of domain blocks of BatA with their templates.

*
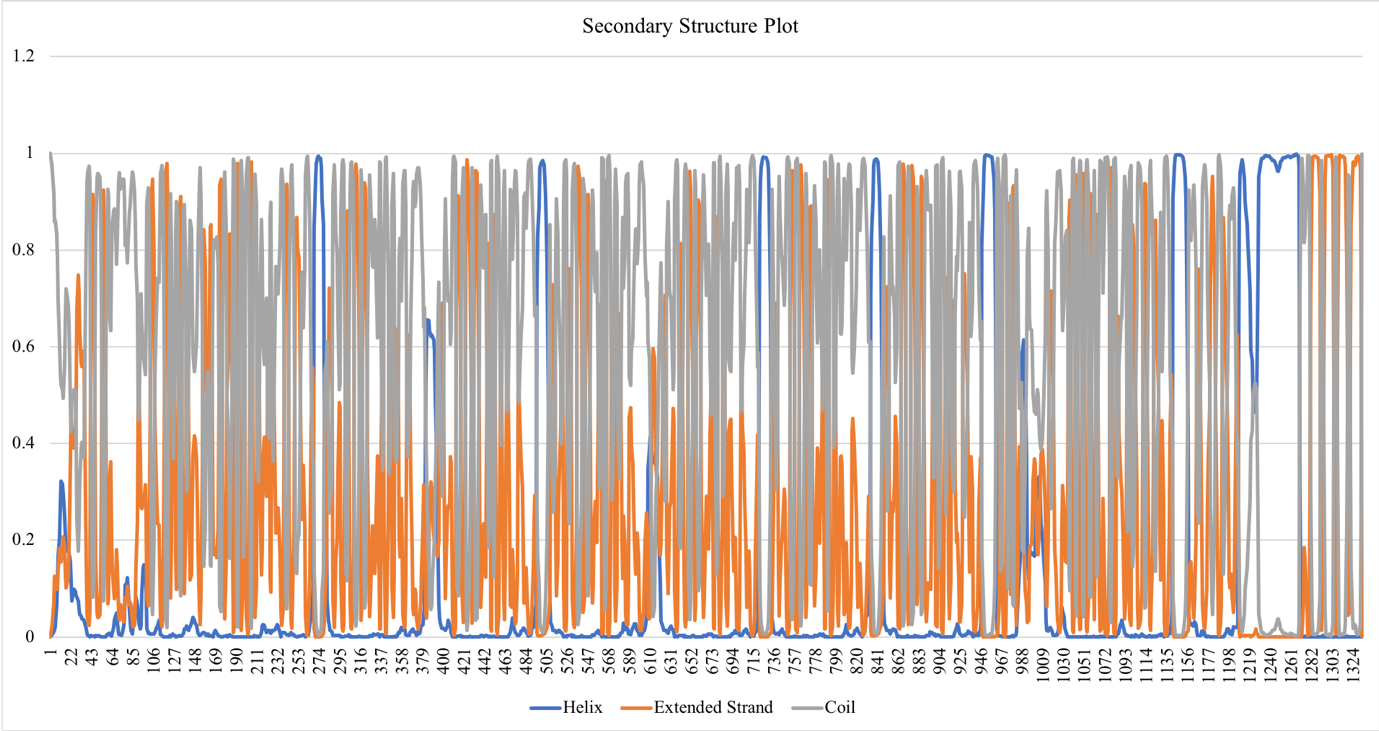
*

**Figure S3.** Secondary structure components of BatA. The horizontal axis is residue number and the vertical axis is probability. three states of secondary structures are presented by different colors in the plot.


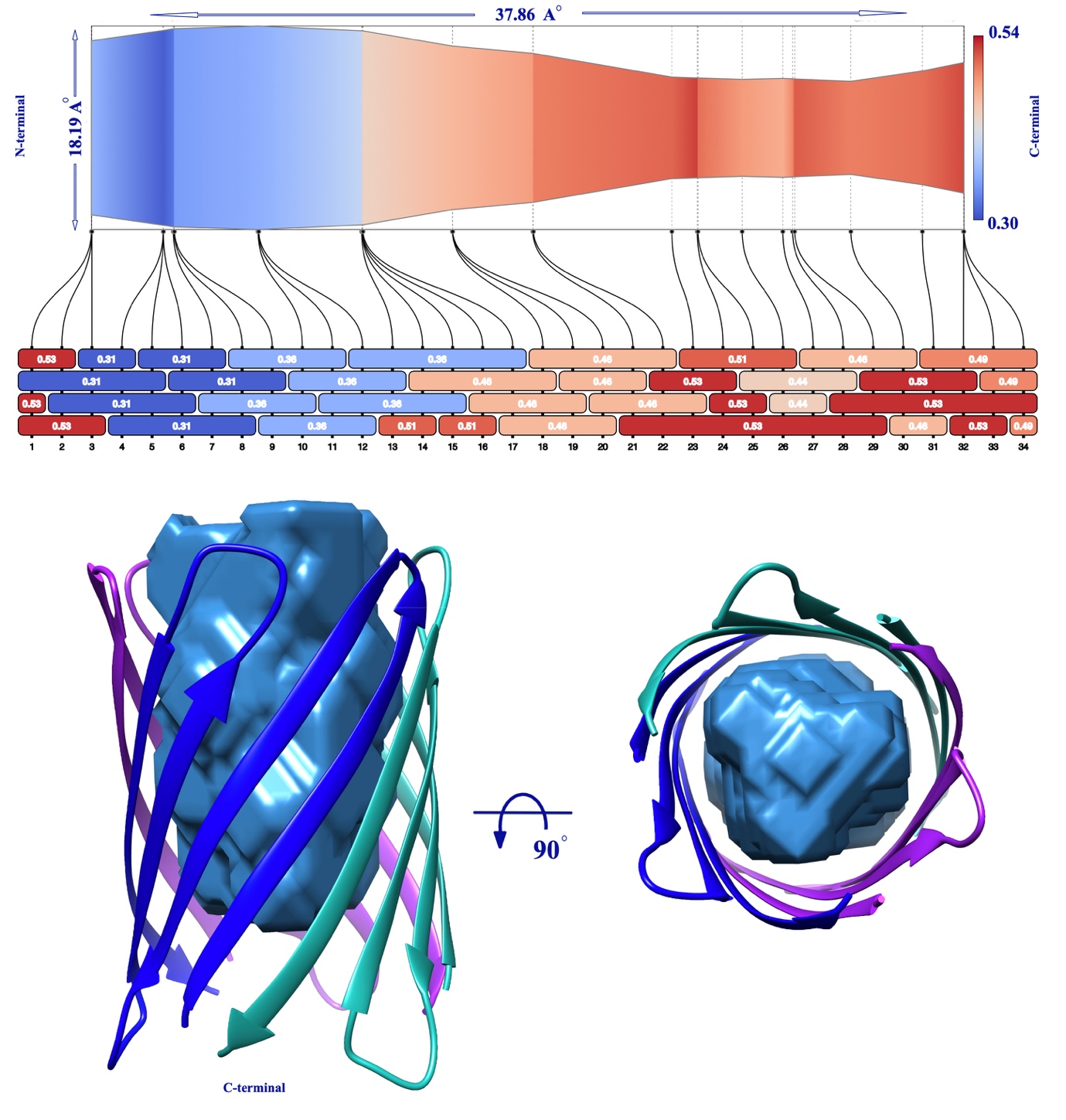


**Figure S4.** Channel properties. 2D presentation of the beta-barrel of BatA is schematically on the top. The top-ranked transmembrane pore is shown using the skin surface (blue).

*
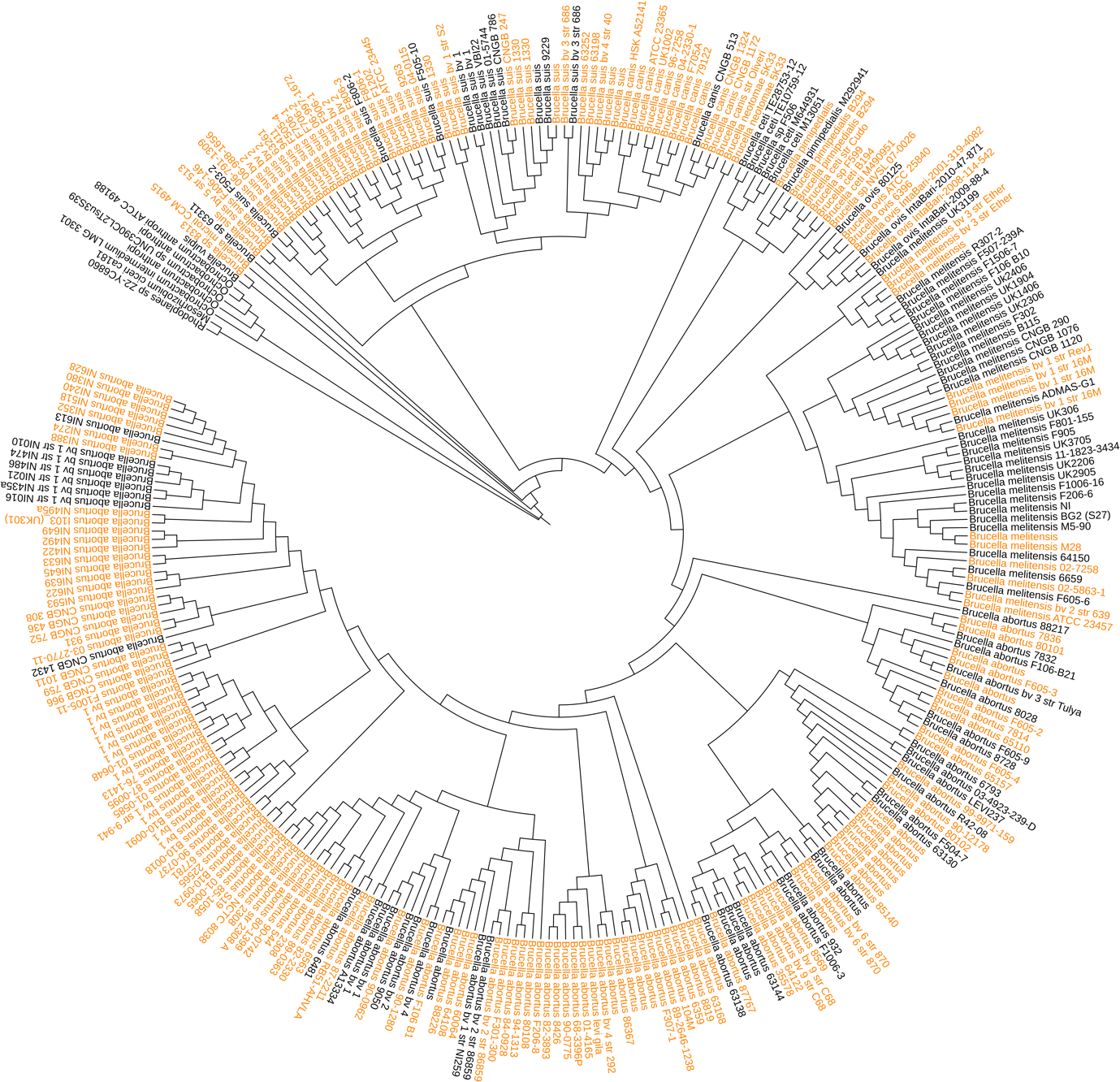
*

**Figure S4.** The phylogenetic tree of Brucellaceae. The TAA encoding genes are present in all 300 leaves of the tree. The orange leaves contain more than one TAA encoding gene.


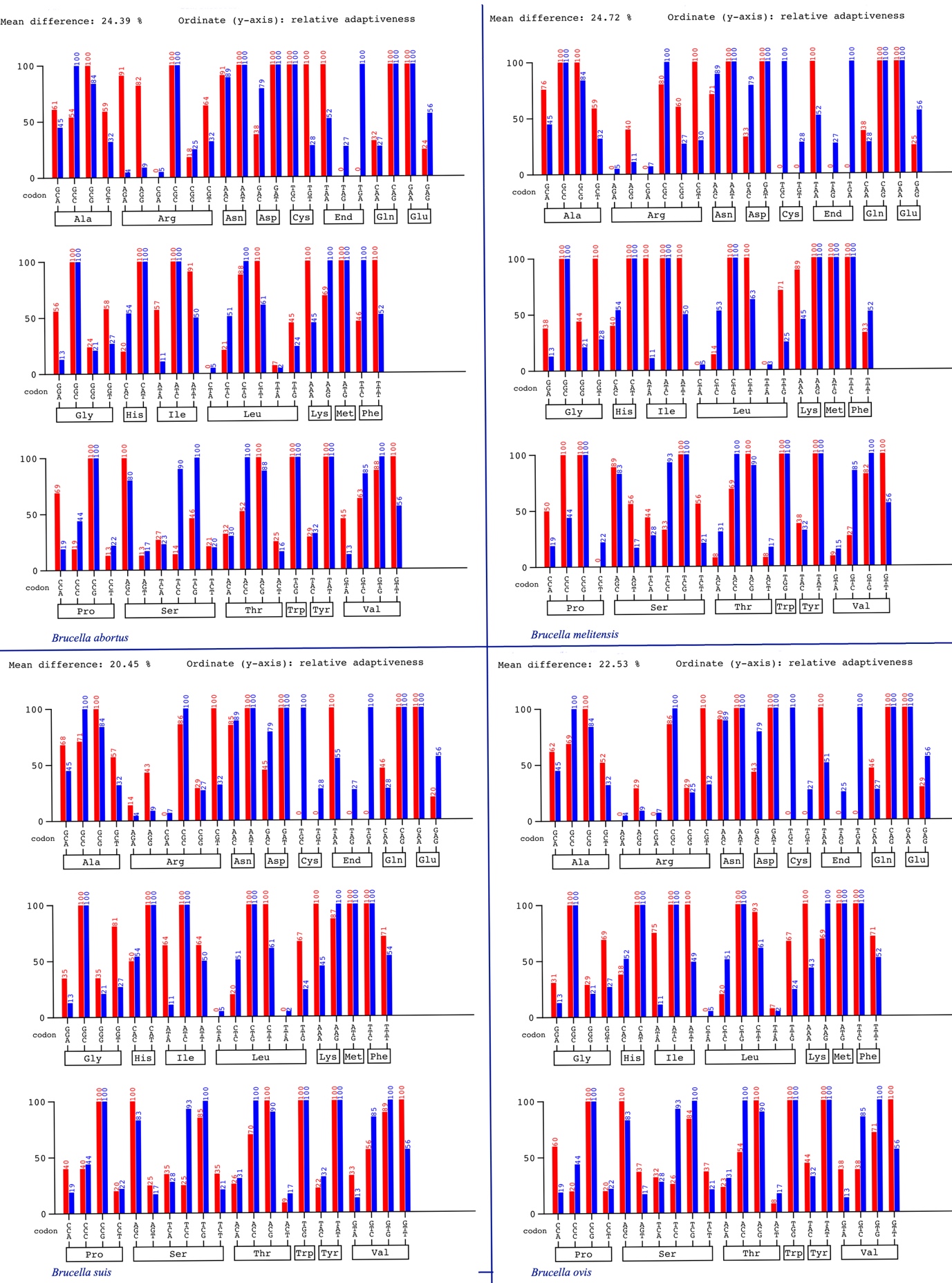

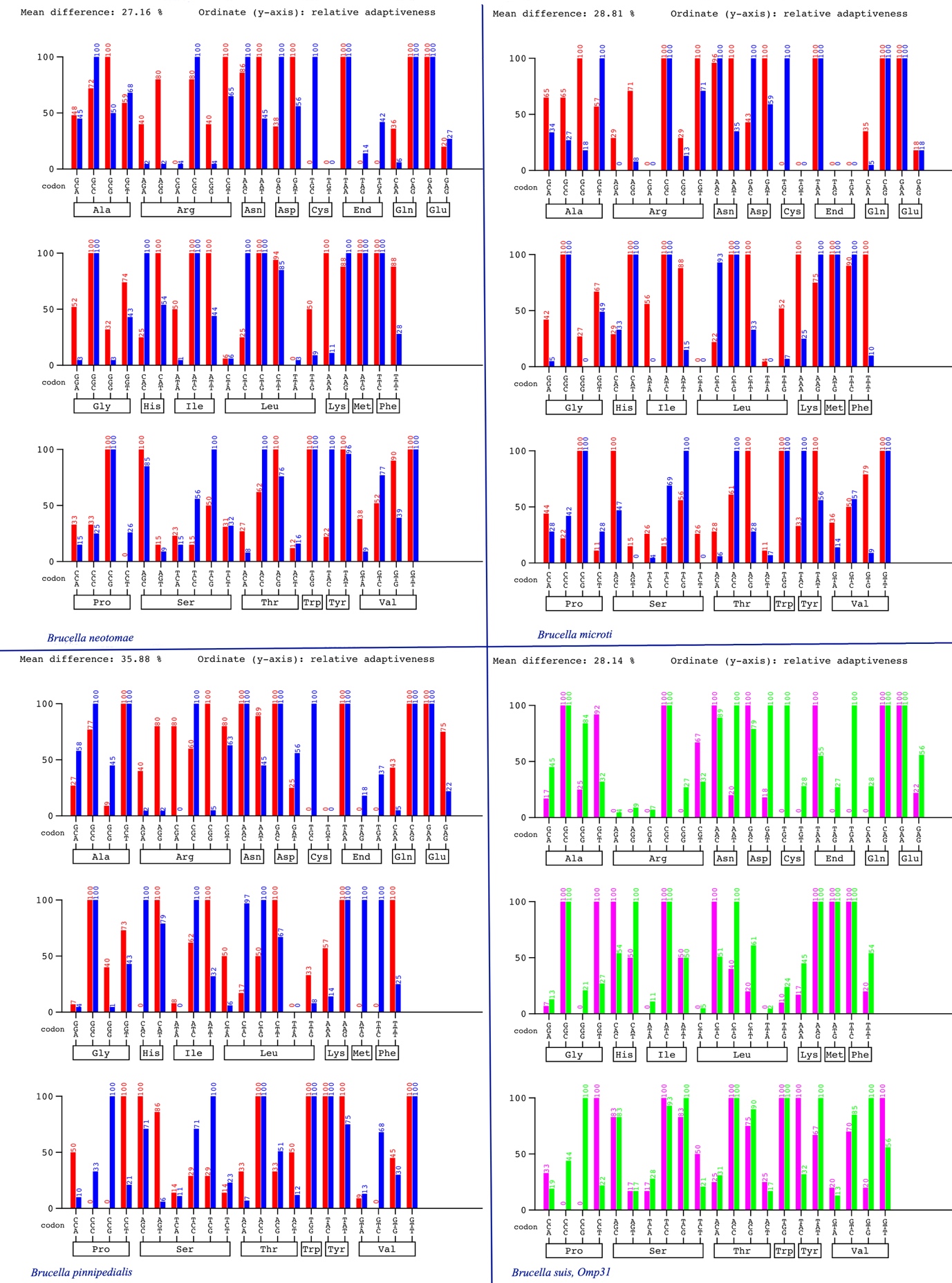


**Figure S5.** The relative codon adaptiveness of TAA encoding genes in comparison to codon adaptiveness of the organism. Red bars are related to the TAA nucleotide sequences and blue bars are calculated based on the codon usage table of the organisms. These values are depicted as cyan and green bars respectively for Omp31 of *B. suis*, as a control*.*

***A short definition of domains observed in the structure of BatA***

In this section, a short definition for each domain is provided. In some cases, the ﻿prominent residue patterns in the domains gave rise to the appellation of domains such as GIN, HIN, Trp-ring, HANS, FGG… Interested readers are referred to the references: (Szczesny and Lupas 2008, Hartmann, Grin et al. 2012, Bassler, Alvarez et al. 2015) for complete information on domain architecture of TAAs.

For each part of the structure of BatA, the monomeric state is presented for simplicity. Structures and related sequences are in the same color.

*
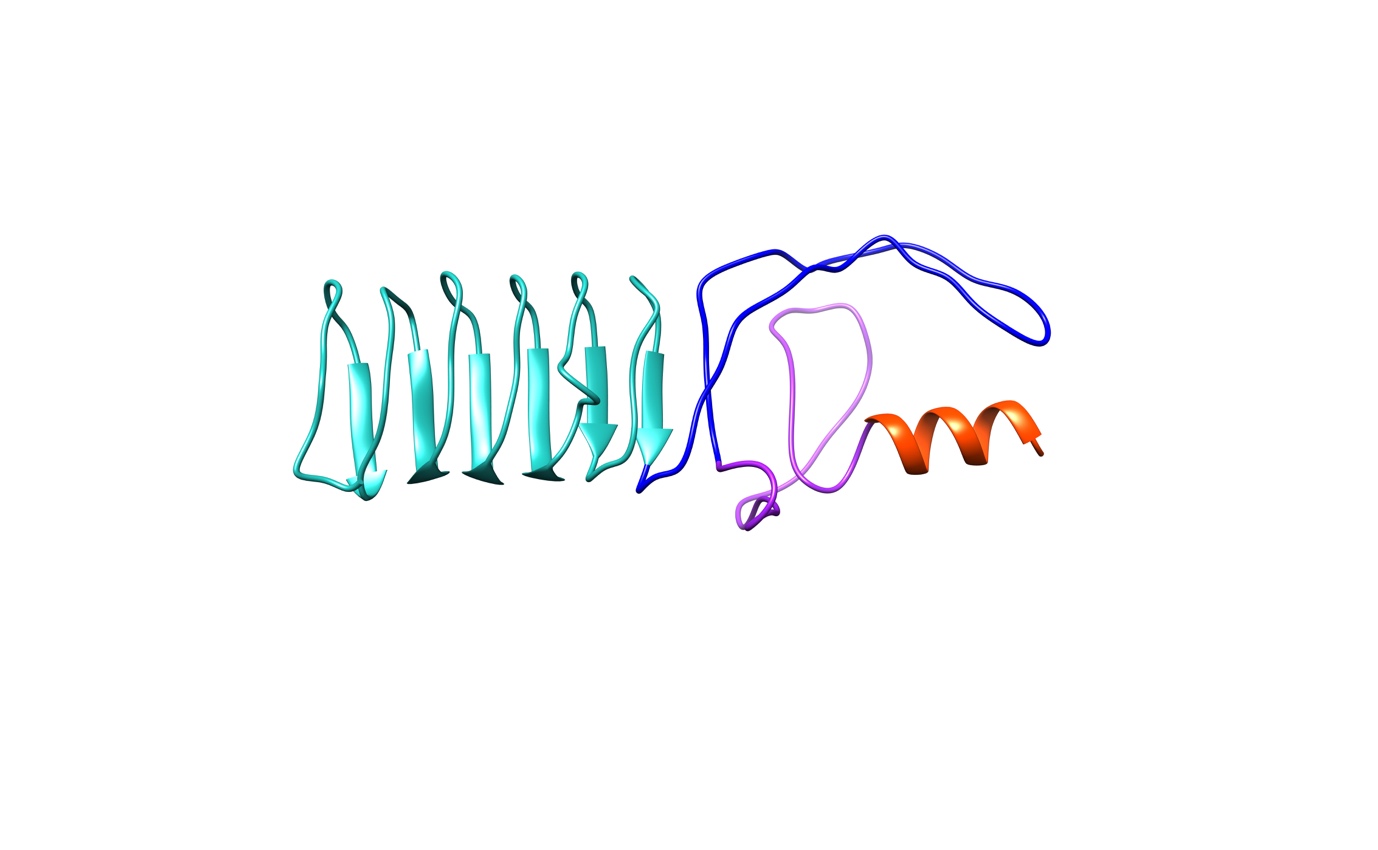
*

**Figure S7:** *C-terminal head followed by HIM, neck, and a short coiled-coil.*

*N-Terminal Head 120-206 FYGNSKEQGSISLTLGGELFVNNGNLGLGGGTDTKAMRIGSMATLTGPSGLRSLAIGAGEIATVASGDDAIAIGTAAQAAHVGSIAL*

*HIM: 207-242*

*GLQSTTELPSLVKDVTINGIKLSAFAGSNPASVLSI*

*Neck:243-271*

*GNDTLKRSITNVGAGRVSKDSTDAVNGSQ*

*Coiled-coil: 272-281*

*LYAVAEQATL*

*Head domains*

N-terminal head and C-terminal head domains are identified as low complexity regions in the dotplot matrix. YadA like head domains are left-handed beta roll, common in TAA architectures (Bassler, Alvarez et al. 2015).

Head domains are divided into two groups viz. interleaved heads, parallel to the fiber axis; and transversal heads perpendicular to the fiber axis. Therefore, YadA like heads are transversal heads (Fig S7).

*HIM*

HIM stands for head insert domain, this domain inserts within the last Yhead repeat before the neck (Szczesny and Lupas 2008). this small domain extends downwards over the neck and mainly composed of coils (Fig S7).

*Neck connectors*

Neck connectors are beta to alpha connectors. the secondary structure of these domains is a beta-strand. The domain divided into various variants viz. short neck, long neck, insert neck, KG, and DALL. Neck domains are the most conserved part of TAAs (Hoiczyk, Roggenkamp et al. 2000). DAVN motif can be found in neck connectors. All sorts of necks were observed in the sequence of BatA except for KG.

*
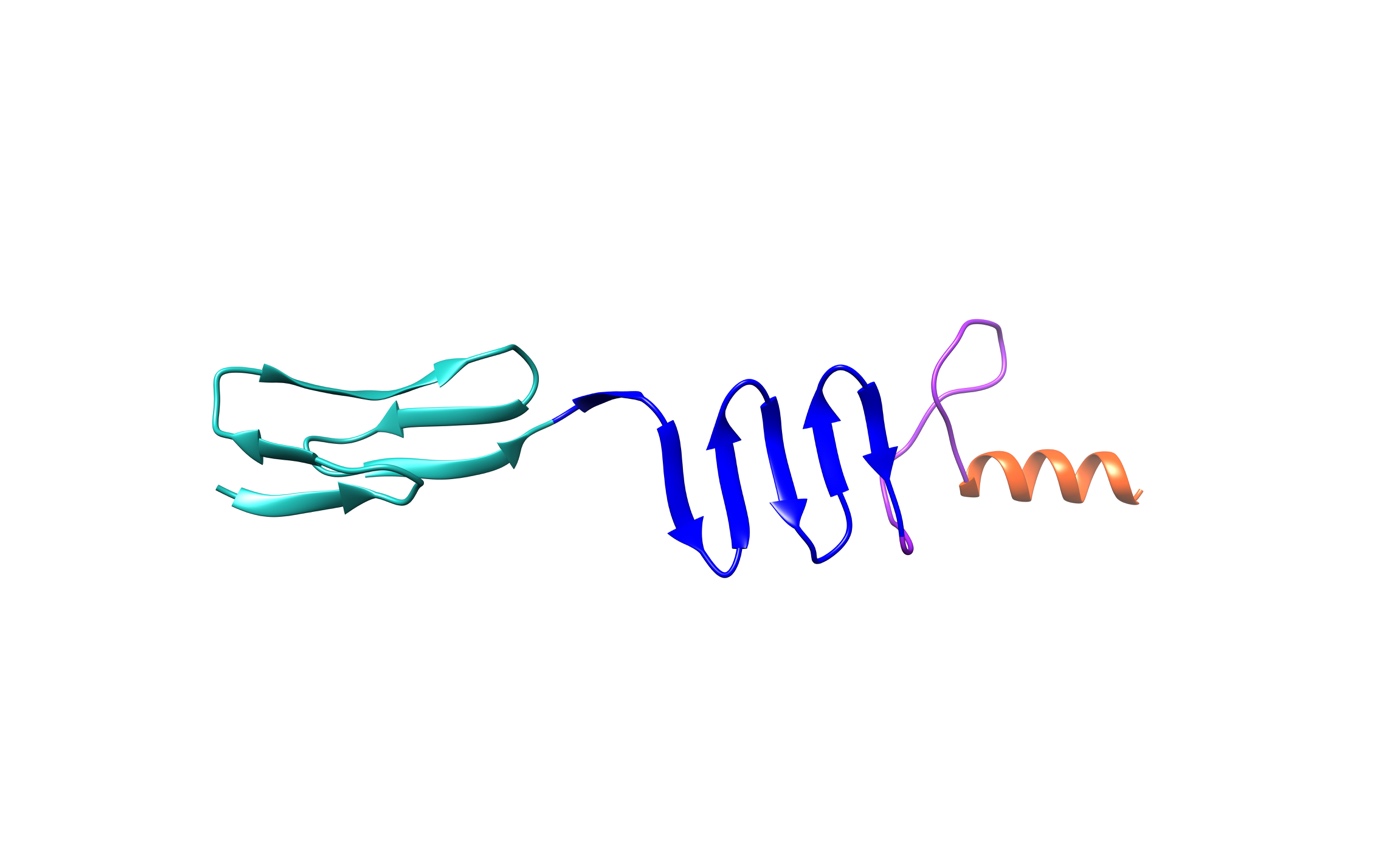
*

*
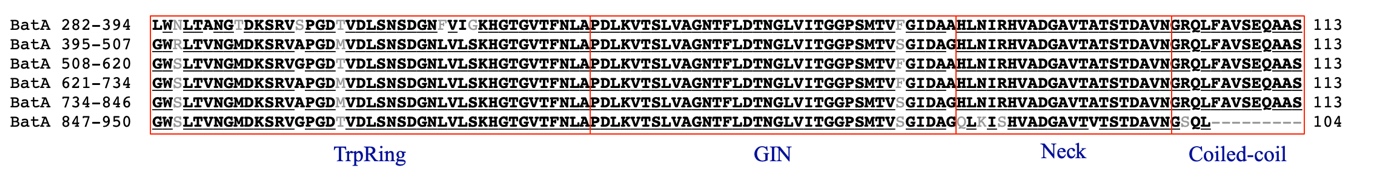
*

**Figure S8:** The core domain of repeated modules is evident in the sequence which is related to the tripartite architecture of TrpRing-GIN-Neck (are appeared to be crucial repeated modular units) domain which is perfectly and tandemly repeated six times within the sequence.

*TrpRing*

A conserved tryptophan residue at the N- terminal of the triplicate architecture at the beginning of this domain give rise to the name TrpRing (Szczesny, Linke et al. 2008). TrpRing is a common interleaved head domain, parallel to the fiber axis (Fig S6).

TrpRing generally transited to the GIN domain (Nominated due to the existence of three residues namely ﻿Gly-Ile-Asn at the C terminal).

*GIN*

GIN domain is perpendicular to the fiber axis, therefore is a traversal head.

This sort of heads exclusively occurred after the interleaved head. In this domain GIN is a single antiparallel beta-sheet forms a triangular beta prism. GIN and Yheads are analogs. TrpRing and GIN connect to a short coiled-coil with the mediation of the neck connector (Fig S8).

After this core domain, there is another YadA like head at the C-terminal. The region is composed of FGG-HANS-HIM-Head and connected to a narrower coiled coil by a neck connector domain.

*
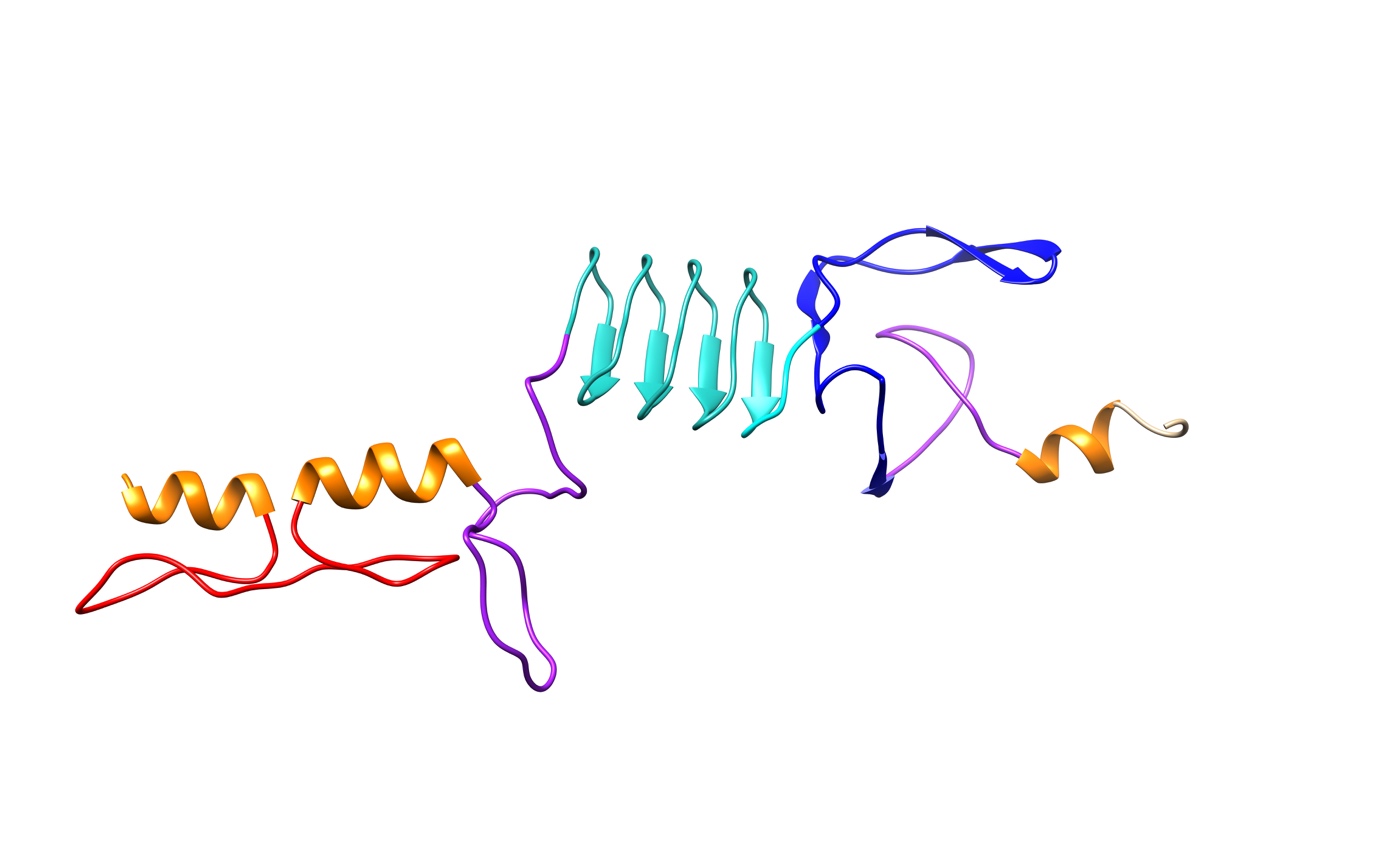
*

**Figure S9:** FGG inserted to the coiled-coil and connects to YadA like head domain by HANS motif. The YadA like head is followed by HIM, Neck, and coiled-coil.

*Head: 1028-1080*

*RALGTNSIAVGSDSVASGEGSISVGNGAQASAHGSVALGENAAAPDANSVALG*

*HIM: 1081-1124*

*AGSKTSEVVATKGTTINGQYYDFAGDAPSGTVSVGDKGAERTIT*

*Neck: 1125-1143*

*NVAAGRISVESTDAVNGSQ*

Coiled coils: 953-997

VAHTIAEHLGGDAHVNADGSVIGPQYTVQKKRYKTIYDAFGGVDE

HANS: 998-1027

NLSNINDILHDIESGGGIKYFHANSIGADS

*FGG*

FGG is a 3-stranded beta meander inserted into the coiled-coil region (Fig S9).

*HANS*

Is abrupt transition of the stalk to the YLHead (alpha to beta connector) the short beta-hairpin interacts with the YLHead. The consensus sequence of KYFHANS is evident in this motif. (in BatA: 1016-KYFHANS-1022, Fig S7)

Finally, the protein ended in a Membrane anchoring region (Fig S8). The membrane channel in the membrane anchoring region is connected to the stalk by a coiled-coil domain.


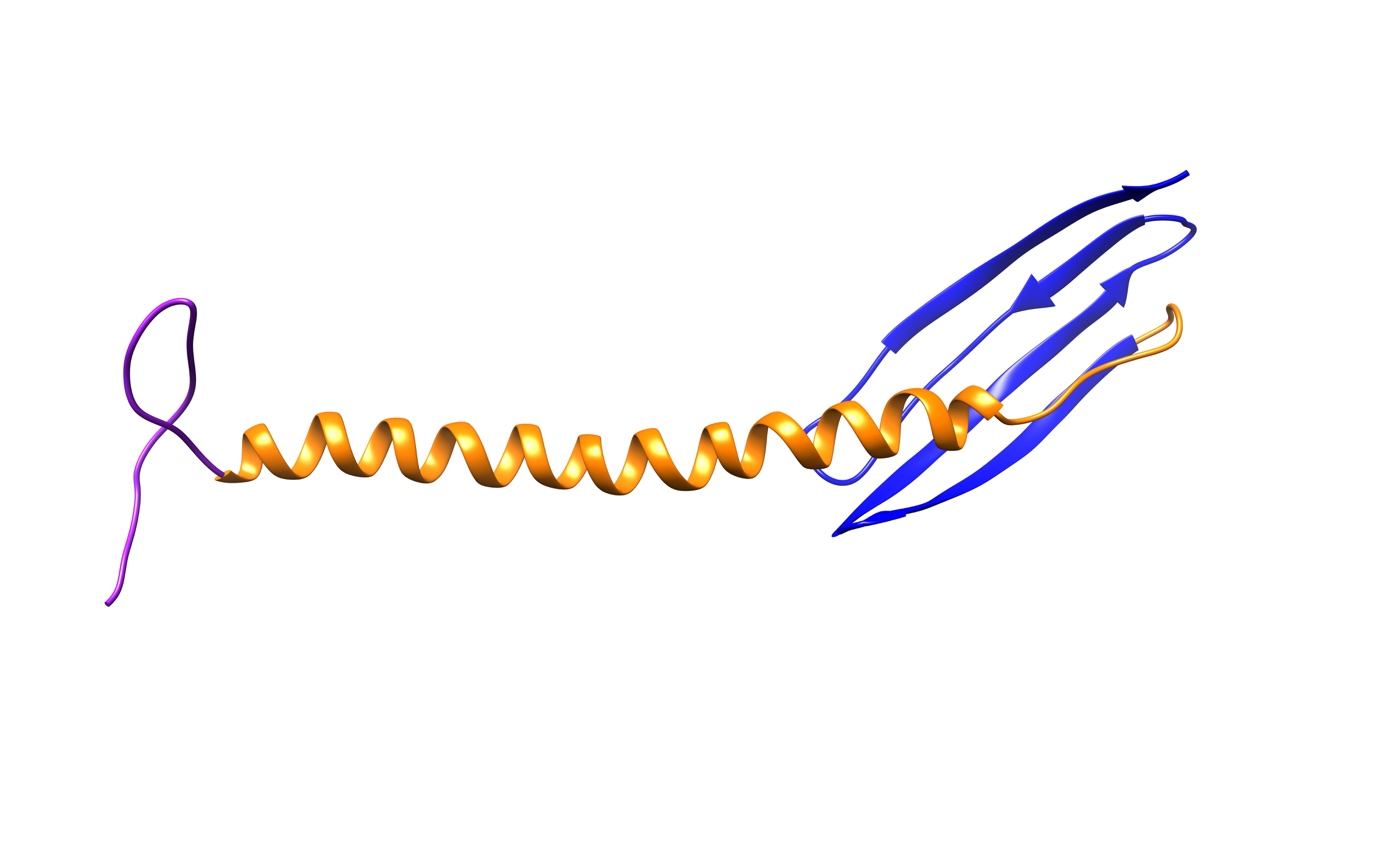


**Figure S10**: Membrane anchoring region and the traversed coiled-coil.

Neck: 1191-1207

*VVLANVADGVHKNDAVN*

Coiled-Coil

VSQLKTLDQANAYTDKKFGKLNEDIVATRIEARQAAAIGLAAASLRYDDRPGK

Channel: 1281-1333

ISAAIGGGFWRGEGAVALGLGHTSEDQRMRSNLSAATSGGNWGMGAGFSYTFN

*Membrane Anchoring domain*

﻿The β-barrel has a central channel that is traversed by three N-terminal α-helices, one from each subunit. The membrane anchoring region is present in all TAAs thus any TAA can be primarily identified by this domain. The betta barrel consists of 12 beta-sheets 4 beta-sheets donated by each monomer.

**Table S1:** Statistical view of BTAAs; number of sequences, sequence length range, and detected signal peptides.

| **Species** | **Hit No.** | **number of unique sequences** | **Sequence length range** | **Signal peptide ~%60*** | **Signal peptide ~%100*** | **Signal peptide** |
| --- | --- | --- | --- | --- | --- | --- |
| *B. abortus* | 285 | 65 | 169-1559 | (95/285) | (8/285) | 36% (103/285) |
| *B. suis* | 90 | 19 | 239-853 | (43/90) | (5/90) | 43 |
| *B. melitensis* | 60 | 11 | 220-1333 | (1/60) | (10/60) | 11 |
| *B. canis* | 31 | 2 | 53 & 613 | (25/31) | - | 25 |
| *B. ovis* | 11 | 5 | 215, 599, & 613 | - | 2 | 2 |
| *B. ceti* | 10 | 5 | 424, 482, 724, 839 | (5/10) | - | 5 |
| *B. neotomae* | 4 | 4 | 267, 482, 643 | - | 1 | 1 |
| *B. pinnipedialis* | 6 | 2 | 278 | - | - | 0 |
| *B. microti* | 2 | 2 | 86, 839 | 1 |  | 1 |
| *Brucella sp* | 10 | 9 | 154, 837 | 3 | 1 | 4 |

*The numbers show the confidence level.

Bassler, J., et al. (2015). "A domain dictionary of trimeric autotransporter adhesins." International journal of medical microbiology **305**(2): 265-275.

Hartmann, M. D., et al. (2012). "Complete fiber structures of complex trimeric autotransporter adhesins conserved in enterobacteria." Proceedings of the National Academy of Sciences **109**(51): 20907-20912.

Hoiczyk, E., et al. (2000). "Structure and sequence analysis of Yersinia YadA and Moraxella UspAs reveal a novel class of adhesins." The EMBO journal **19**(22): 5989-5999.

Sankarasubramanian, J., et al. (2016). "BrucellaBase: genome information resource." Infection, Genetics and Evolution **43**: 38-42.

Szczesny, P., et al. (2008). "Structure of the head of the Bartonella adhesin BadA." PLoS Pathog **4**(8): e1000119.

Szczesny, P. and A. Lupas (2008). "Domain annotation of trimeric autotransporter adhesins—daTAA." Bioinformatics **24**(10): 1251-1256.
